# Efficient Extraction of Nanoplastics from Aqueous Media Using Natural Deep Eutectic Solvents: Experimental and Molecular Insights

**DOI:** 10.1021/acsomega.6c02548

**Published:** 2026-08-18

**Authors:** Francisco Thálysson Tavares Cavalcante, Lucas Lima Bezerra, Norberto de Kássio Vieira Monteiro, Rilvia Saraiva de Santiago-Aguiar

**Affiliations:** † Department of Chemical Engineering, Center for Technology, Federal University of Ceara, Campus do Pici, bl. 709, Fortaleza, Ceará 60455-760, Brazil; ‡ Department of Analytical and Physical Chemistry, Center of Sciences, Federal University of Ceara, Campus do Pici, bl. 940, Fortaleza, Ceará 60440-900, Brazil

## Abstract

Microplastics and nanoplastics are persistent contaminants of global concern due to their ubiquity in aquatic systems and their potential ecological and toxicological impacts. Conventional solvent-based extraction methods are often inefficient or environmentally harmful, emphasizing the need for greener alternatives. In this study, natural deep eutectic solvents (NADES) composed of thymol (Thy), menthol (Men), and fatty acids were prepared and evaluated for the extraction of polystyrene (PS) nanoplastics from aqueous media. Extraction experiments were performed at 25 °C under controlled agitation, and the influence of pH (3.0–9.0) and solvent composition on extraction efficiency was systematically investigated. The Thy:Men (1:1) system exhibited the highest extraction efficiencies of 97.14 ± 0.49%, 75.35 ± 20.90%, and 78.19 ± 1.36% at pH 3.0, 7.0, and 9.0, respectively, reaching equilibrium after approximately 2 h. Molecular dynamics (MD) simulations were employed to elucidate the molecular mechanisms underlying the extraction process. The results revealed that menthol molecules preferentially accumulate at the water–NADES interface, forming hydrogen bonds with water while engaging in van der Waals interactions with PS. Thymol contributes primarily through π–π stacking interactions with the aromatic backbone of PS, resulting in strong stabilization within the NADES phase.

## Graphical abstract

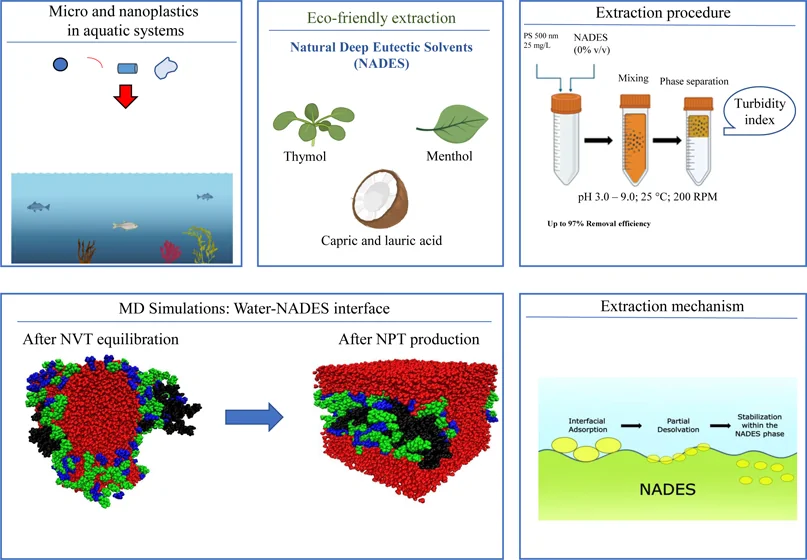

## 1 Introduction

The pervasive presence of plastic-derived micro- and nanomaterials in the environment has emerged as a major global concern. Microplastics (<5000 μm, >1 μm) and nanoplastics (<1000 nm) are ubiquitous contaminants widely distributed across the environment - found in air, water, soil, and sediments.^1,2^ They originate from the degradation of diverse plastic materials used in daily human life, including cosmetics, abrasives, pellets, packaging materials, construction debris, agricultural materials, tire wear, and others.^3^ Given the global demand for these materials for these materials and their impacts, which are not yet fully understood, raising concerns underscores the urgent need for effective monitoring and mitigation strategies.

While research interest continues to grow, the analysis of micro- and nanoplastics remains a significant challenge, and it is particularly pronounced for nanoplastics, whose small size, tendency to aggregate, and interactions with complex matrices make their detection, isolation, and quantification especially difficult. Addressing these issues requires integrated analytical approaches tailored to the unique properties of these contaminants, along with the development of standardized and cross-study validated protocols.

One strategy that has been gaining attention is the use of deep eutectic solvents (DES) to separate nanoplastics from water, as previously described in the literature. DESs are eutectic mixtures composed of a hydrogen bond donor (HBD) and a hydrogen bond acceptor (HBA), in which strong, specific intermolecular interactions lead to nonideal behavior, resulting in a eutectic point temperature lower than that predicted for an ideal liquid mixture and a significant depression of the melting temperature relative to the individual components.^4^ These solvents offer significant advantages over traditional organic solvents, including biodegradability, low toxicity and tunable physicochemical properties. Among them, natural deep eutectic solvents (NADES) are produced from the mixture of nontoxic natural materials, such as organic acids, sugars, and monocyclic monoterpenoids. NADESs with hydrophobic properties have been used to remove metal ions, furfural, and contaminants from water. The possibility of designing solvents with high hydrophobicity makes them suitable for the removal of nonpolar polymers such as poly­(ethylene terephthalate) (PET), PS, and polylactic acid (PLA) from water, as first proposed by Hunter et al.^5^

Computational chemistry methods, such as molecular dynamics (MD) simulations, have proven instrumental in elucidating interactions between micro/nanoplastics and extractants, such as alternative solvents and adsorbent materials, at the molecular level.^6–18^ The computational investigation of the interaction between nanoplastics and NADES in an aqueous environment should provide valuable insights, serving as a complementary analysis to determine the best extraction solvent. This study is proposed as a cross-validation study of the potential of NADES as extractants of different nanoplastic particles from water. The interpretations of the simulations performed can be used to guide the design of hydrophobic NADES to remediate plastics from aqueous systems.

## 2 Materials and Methods

### 2.1 Materials

Menthol (Men, ≥98.5%) and dodecanoic acid (C_12_, ≥98%) were purchased from Êxodo Científica; thymol (Thy ≥99%), and decanoic acid (C_10_, ≥98%) were purchased from Neon. Polystyrene (PS) microspheres (500 nm) were obtained from Sigma-Aldrich. A 10% (v/v) HNO_3_ solution was prepared from nitric acid (HNO_3_, 65%) obtained from Chem-Lab (Belgium). Deionized water was used throughout the experiments.

### 2.2 Experimental Setup

#### 2.2.1 Preparation of the Natural Deep Eutectic Solvents

Four NADESs, namely, C_10_:Men 1:2, C_12_:Men 1:2, Thy:Men 1:1, and Thy:Men 1:2, and one eutectic mixture (ES), namely, C_10_:C_12_ 2:1, were prepared by mixing the components in the specified molar ratios of components, followed by heating and stirring at 80 °C until a homogeneous liquid was obtained. Each NADES is denoted according to its components and molar ratio; for example, Thy:Men (1:1) indicates equal molar amounts of thymol and menthol (Table 1). The molar ratios were selected based on previous studies involving the extraction of compounds from aqueous systems using hydrophobic NADES, in which these compositions demonstrated favorable phase behavior and extraction performance.^6,19–22^ The resulting NADESs were stored in sealed bottles inside a desiccator at room temperature until use.

**Table 1:** Composition, Mole Fractions, Molar Mass, and Purity of the Eutectic Solvent (ES) and Natural Deep Eutectic Solvents (NADES) Prepared^a^

Solvent	HBA	HBD	HBA molar mass (g mol^–1^)	HBD molar mass (g mol^–1^)	*x*(HBA)	*x*(HBD)	molar mass NADES (g mol^–1^)
C_10_:C_12_ (2:1, ES)	decanoic acid	dodecanoic acid	172.27	200.32	0.667	0.333	181.61
C_10_:Men (1:2)	decanoic acid	menthol	172.27	156.27	0.333	0.667	161.60
C_12_:Men (1:2)	dodecanoic acid	menthol	200.32	156.27	0.333	0.667	170.95
Thy:Men (1:1)	thymol	menthol	150.22	156.27	0.500	0.500	153.23
Thy:Men (1:2)	thymol	menthol	150.22	156.27	0.333	0.667	154.25

a Molar mass (M) of ES and NADES were calculated as weighted average based on the molar ratio of HBA and HBD: *M*_ES,NADES_ = (*x*_HBA_ × *M*_HBA_) + (*x*_HBD_ × *M*_HBD_). Purity values are as provided by the suppliers. C_10_ = decanoic acid; C_12_ = dodecanoic acid; Men = menthol; Thy = thymol.

#### 2.2.2 Nanoplastics Extraction Using NADES

Extraction experiments were conducted by introducing 1.5 mL of NADES (top phase) into 15 mL of the aqueous phase containing PS nanoplastics (bottom phase) in a 50 mL Erlenmeyer flask at room temperature. The mixtures were stirred using a refrigerated incubator with orbital shaking (TE-424, TECNAL) at 200 rpm, at 25 °C. The initial nanoplastic concentration in the aqueous phase was 25 mg/L in deionized (DI) water.

After predetermined time intervals, the mixtures were transferred to separation funnels and allowed to stand for 20 min to achieve complete phase separation, which was visually confirmed by the formation of two distinct phases and a clear interface. Preliminary experiments indicated that no further changes in turbidity occurred after this period, suggesting that equilibrium had been reached. Samples (10 mL) were collected from the aqueous phase and their turbidity was measured with a HI98703 portable turbidimeter. The readings were converted to concentration using two calibration curves constructed from a series of solutions prepared by dilution of a stock solution (Figure S1, in the Supporting Information). The first curve covered 1–10 mg L^–1^ (1, 2, 5, 7.5, and 10 mg L^–1^) and the second covered 10–25 mg L^–1^ (10, 12, 15, 20, and 25 mg L^–1^). The overall extraction experiment lasted 16 h.

Extraction efficiency (% extracted) was calculated by comparing the measured concentration at each time point with the initial concentration. To describe the efficiency over time, the data were fitted to a hyperbolic function using nonlinear regression. The relationship is expressed in eq 1

1 $$y=a\frac{x}{b+x}$$

where *y* is the percentage of plastic particles extracted, *x* is the elapsed time since the start of extraction, *a* represents the theoretical maximum fraction of particles extracted, and *b* is the time required to reach half of this maximum. This empirical model resembles the Langmuir adsorption isotherm, often used to describe sorption kinetics.

The limit of detection (LOD) and limit of quantification (LOQ) were determined according to IUPAC^23^ recommendations using the slope of the calibration curve and the standard deviation of ten blank solution turbidity values, according to eqs 2 and 3

2 $$LOD=3.3\times \frac{\sigma }{S}$$

3 $$LOQ=10\times \frac{\sigma }{S}$$

where σ corresponds to the standard deviation of matrix-matched blank signals. The calibration curve exhibited linear behavior in the range of 0.05–2 mg L^–1^ (*R* ^2^ = 0.999). For the PS nanobeads, the LOD and LOQ were calculated as LOD = 0.02 mg L^–1^ and LOQ = 0.067 mg L^–1^. These values confirm the suitability of the method for the quantification of NPs in the tens of μg L^–1^ range.

To determine the influence of pH values, 0.1 M hydrochloric acid and 0.1 M sodium hydroxide solutions were used to adjust the pH value (from 3 to 9) of the nanoplastic solutions. Then, the procedure to determine the nanoplastic concentration was the same as described above. After obtaining the best solvent and extraction pH, the extraction progress was monitored under these conditions by determining the hourly yield for up to 7 h.

### 2.3 Contamination Control

Effective quality control is essential for reliable nanoplastic analysis, particularly regarding airborne particles and polymer-based equipment. Best practices include the use of nonpolymer tools whenever possible, clean laboratory environments, and the use of field and procedural blanks to quantify potential external sources of contamination.^24,25^

All experimental work was performed by personnel wearing clean cotton laboratory coats and sterile gloves. All glassware was cleaned by soaking overnight in 10% nitric acid, followed by triple rinsing with distilled water.^26,27^ Nonglassware materials were rinsed with ethanol and subsequently with distilled water.^28^ Only single-use polymeric pipet tips were employed to minimize cross-contamination, and all sample containers were covered immediately after preparation to prevent airborne exposure. Replicate experiments and procedural blanks confirmed that background contamination was negligible and did not affect the analytical results.^29,30^

### 2.4 Computational Details

#### 2.4.1 Nanoplastic Modeling

Considering that shorter-chain polymers are more likely to form low-melting glasses and liquids, and that low molecular weight hydrophobic polymers are more prone to interact with the hydrophobic region of lipid membranes, leading to cell damage,^31^ an isotactic polystyrene (PS) chain containing 20 repeat units (Figure S2A) was built to represent a model nanoplastic fragment Polymer structure was constructed using Avogadro 2, version 1.99.0,^32^ resulting in a chain containing 40 carbon atoms in the main backbone.^33^ Subsequently, all geometries were optimized using quantum chemical methods, as described below.

#### 2.4.2 Quantum Calculations

Three-dimensional structures of thymol (Figure S2B), menthol (Figure S2C), decanoic acid (Figure S2D), and dodecanoic acid (Figure S2E) were optimized in the gas phase at the Density Functional Theory (DFT) level of theory using the hybrid B3LYP functional^34–36^ and the 6-311+G­(d,p) basis set,^37^ implemented in the Gaussian 09 software.^38^ The isotactic polystyrene chain with 20 repeat units was optimized in the gas phase using the semiempirical PM6 method,^39^ implemented in Molecular Orbital PACkage (MOPAC).^40^ The absence of imaginary vibrational frequencies confirmed that all optimized geometries were not transition states.^41^

#### 2.4.3 Molecular Dynamics Simulations

All MD simulations were performed using GROMACS, version 2023.3 ^42^ implemented with Optimized Potentials for Liquid Simulations—All Atom (OPLS-AA) force field.^43^ Force field parameters were generated using Polymer Parameter Generator (PolyParGen)^44^ for the polymeric chain and Ligand Parameter Generator (LigParGen)^45–47^ for the other molecular species described in Section 2.1. Nonbonded interactions were represented as the sum of short-range Lennard-Jones 12-6 potential and long-range Coulombic potential. Bonded interactions included bond, angle, and dihedral potentials, following the definitions provided by the force field.^48^

Simulation boxes of 9 nm × 9 nm × 9 nm were constructed for the molecular dynamics simulation. To represent the behavior of the polystyrene nanoplastics in the water, 10 PS (20-mer) chains were randomly inserted using Packmol^49^ and filled with water in the TIP3P solvation model.^50^ Energy minimization was performed using the steepest descent^51^ and conjugate gradient^52^ algorithms until the energy convergence criterion of 10 kJ mol^–1^ nm^–1^ was reached. Energy minimization and subsequent refinement steps allow the system to relax toward local minima of the potential energy surface defined by the force field.^53,54^ After that, the system was equilibrated for 1 ns in the Number of particles (N), Volume (V), Temperature (T) NVT ensemble using a V-rescale thermostat^55^ at 303.15 K followed by 1 ns in the Number of particles (N), Pressure (P), Temperature (T) NPT ensemble with a C-rescale barostat at 303.15 K and 1 bar.^56^ Finally, a production step was performed under 50 ns NPT conditions at 303.15 K, at 1 bar. The Leap–Frog algorithm^57^ was applied to integrate the motion equation with a time step of 2.0 fs. The long-range interactions were modeled using particle mesh Ewald sum (PME) method with a cutoff of 1 nm.^58^ The Linear Constraint Solver (LINCS) algorithm^59^ was utilized to restrict all bonds.

To evaluate the extraction potential of the NADES, the PS-water boxes were expanded by 0.2 nm, the minimal size required to insert the NADES component molecules. The molar ratios were consistent with experimental batches, corresponding to 150 thymol and 150 menthol molecules (Thy:Men 1:1), 100 thymol and 200 menthol (Thy:Men 1:2). After NADES insertion, systems were reminimized (steepest descent, 10 kJ mol^–1^ nm^–1^ tolerance), equilibrated for 1 ns NVT (V-rescale thermostat) and 10 ns NPT (C-rescale barostat), followed by a 200 ns NPT production at 298 K and 1 bar. All other simulation parameters were identical to those described previously, except that in this stage, the LINCS algorithm^59^ was applied only to constrain hydrogen bonds.

The Interaction Potential Energy (IPE) [eq 4] was used to quantify the affinity between the system components in pair combinations (PS–water, PS–NADES, and water–NADES). This energy is obtained by the sum of the short-range Lennard-Jones and Coulomb energies. The N_i_ and N_j_ terms correspond to the total number of atoms of species i and j, respectively, while V_vdW_ and V_elec_ denote the van der Waals forces and electrostatic energy contributions, respectively.^60^ All simulations were performed in duplicate as independent replicas, using identical simulation parameters and geometry. This approach was adopted to ensure the reproducibility of the results and to verify that the observed aggregation behavior is not dependent on a specific initial configuration.^61–63^

4 $$IPE_{i,j}=\sum_{i}^{N_{i}}V_{vdW}\left(r_{ij}\right)+\sum_{j\neq i}^{N_{j}}V_{elec}\left(r_{ij}\right)$$

## 3 Results and Discussion

### 3.1 Nanoplastics Extraction with NADES

Figure S3 shows the optimized structures of the molecules used in the molecular dynamics simulations. The extraction performance of the NADES at different pH values was evaluated based on their extraction efficiencies, as shown in Table 2. The extraction experiments were conducted under the same conditions of 25 °C, 200 rpm, 25 mg/L, and a 10:1 water:NADES volume ratio. No measurable extraction was observed for C_10_:C_12_ (2:1) under any pH condition, with turbidity values higher than those of the blank (no NADES added), indicating phase instability due to the high water content. The observed phase instability and apparent mixture dissociation suggest that the C_10_:C_12_ system does not exhibit the nonideal, structured behavior typically associated with DES, supporting its classification as a simple eutectic mixture.^4^

**Table 2:** Nanoplastic Extraction Efficiencies (%) of the NADESs Over the pH Range 3.0–9.0^a^ ^,^ ^b^

solvent	pH 3.0	pH 4.0	pH 7.0	pH 9.0
C_10_:C_12_ (2:1)	–	–	–	–
C_10_:Men (1:2)	96.23 ± 0.71	74.17 ± 19.24	65.24 ± 13.81	72.87 ± 21.09
C_12_:Men (1:2)	96.29 ± 0.46	66.63 ± 7.39	26.70 ± 21.94	58.93 ± 26.29
Thy:Men (1:1)	97.14 ± 0.49	57.58 ± 7.71	75.35 ± 20.90	78.19 ± 1.36
Thy:Men (1:2)	96.55 ± 0.46	52.12 ± 7.52	56.36 ± 4.14	45.62 ± 10.05

a Experimental Conditions are Detailed in Section 2.2.2.

b Values are reported as mean ± standard deviation (*n* = 3). The C_10_:C_12_ (2:1) system showed turbidity values over 1000 NTU and thus could not be evaluated.

The other NADES showed extraction efficiencies ranging from 26.70 ± 21.94% (C_12_: Men 1:2, pH 7.0) to 97.14 ± 0.49 (Thy:Men 1:1, pH 3.0). The extraction efficiency of PS nanoplastics at pH 7.0 was close to that reported by Zhang, which ranged from 55–75% after around 12 h.^19^ However, the higher value was obtained for the C_10_:Men 1:2, while the lower was obtained for Thy:Men 1:1, although both fall within the high standard deviation observed in this study. The extraction of nanoplastics likely occurs through their transfer from the aqueous phase to the NADES phase, where they are retained at the interface and within the hydrophobic domains. This process is facilitated by agitation, which promotes the formation of small droplets and increases the contact area between phases.

The high extraction efficiency observed for PS can be attributed to favorable intermolecular interactions, particularly hydrophobic effects and π–π interactions between the aromatic rings of thymol and the polymer backbone.^64^ These interactions enhance the affinity of PS for the NADES phase, promoting its partitioning from water. Hydrogen bonding may also contribute, depending on the functional groups present at the particle surface.^6^

In addition, the NADES may partially soften or swell the polymeric structure, reducing its colloidal stability and facilitating its removal from the aqueous phase.^6^ This effect can further enhance the transfer of nanoplastics into the NADES phase.

The experimental observations also suggest that mass transfer plays a critical role in the extraction process. Systems agitated at lower speeds (150 rpm) exhibited negligible extraction efficiency, indicating insufficient dispersion and interfacial area.^65^ Preliminary tests revealed some variability in extraction efficiency among flask positions within the incubator. To minimize this source of experimental variation and ensure reproducible conditions, all subsequent experiments were conducted using the central positions of the incubator, which consistently yielded higher extraction efficiencies.^66^

The results are consistent with those reported by Ishtaweera et al.,^20^ who obtained nearly complete removal of nanoplastic surrogates using Thy:Men (1:1), with extraction efficiencies for PS beads (100–1000 nm) ranging from 96.5 to 100% after 24 h. The authors also reported size-dependent extraction efficiencies for the C_10_:C_12_ (2:1) system, achieving up to 98.6% removal for larger PS particles (0.2–1.0 μm) but significantly lower efficiencies for smaller particles (0.1 μm). The high extraction efficiencies reported are likely influenced by the extraction protocol, particularly the use of intensive mixing to promote emulsification followed by centrifugation to induce phase separation. These conditions can enhance apparent extraction efficiency by promoting physical transfer of particles rather than true thermodynamic partitioning. In contrast, the present study evaluates extraction performance under less forced conditions, where differences in intermolecular interactions and nonideal behavior become more evident.

In contrast to Hunter et al., but in agreement with the present study, the authors observed a consistently excellent efficiency of the Thy:Men 1:1 across all nanoplastic sizes, unlike its 1:2 counterpart, which showed variable performance. For example, in the case of 0.3 μm PS beads, the authors reported a 99.5% extraction rate for the Thy:Men 1:1 system, Thy:Men 1:2 only achieved 67.4%.

The authors concluded that increasing menthol proportion in the NADES formulation lowered extraction efficiency, despite both systems being similarly hydrophobic based on their low water miscibility. This highlights the critical role of intermolecular interactions within the NADES. The authors also observed that some NADESs perform well in extracting nanoplastic at higher concentrations but are less effective below specific thresholds. This behavior was also observed in the initial extraction tests conducted by our group.

Considering the high efficiencies obtained, Thy:Men (1:1) at pH 3.0 was selected as the optimal system. The extraction kinetics are shown in Figure 1. The concentration decreased sharply during the first 2 h, reaching a plateau after 4 h with minimal standard deviation, confirming the rapid and stable extraction capacity of the NADES. In comparison, Hunter et al.^19^ reported maximum PS extraction only after 12 h, and Zhang et al.^6^ achieved similar yields in 15 min using lignin-based NADES. However, neither study evaluated the influence of pH, which proved to be a key factor in the present work.

**Figure 1:**
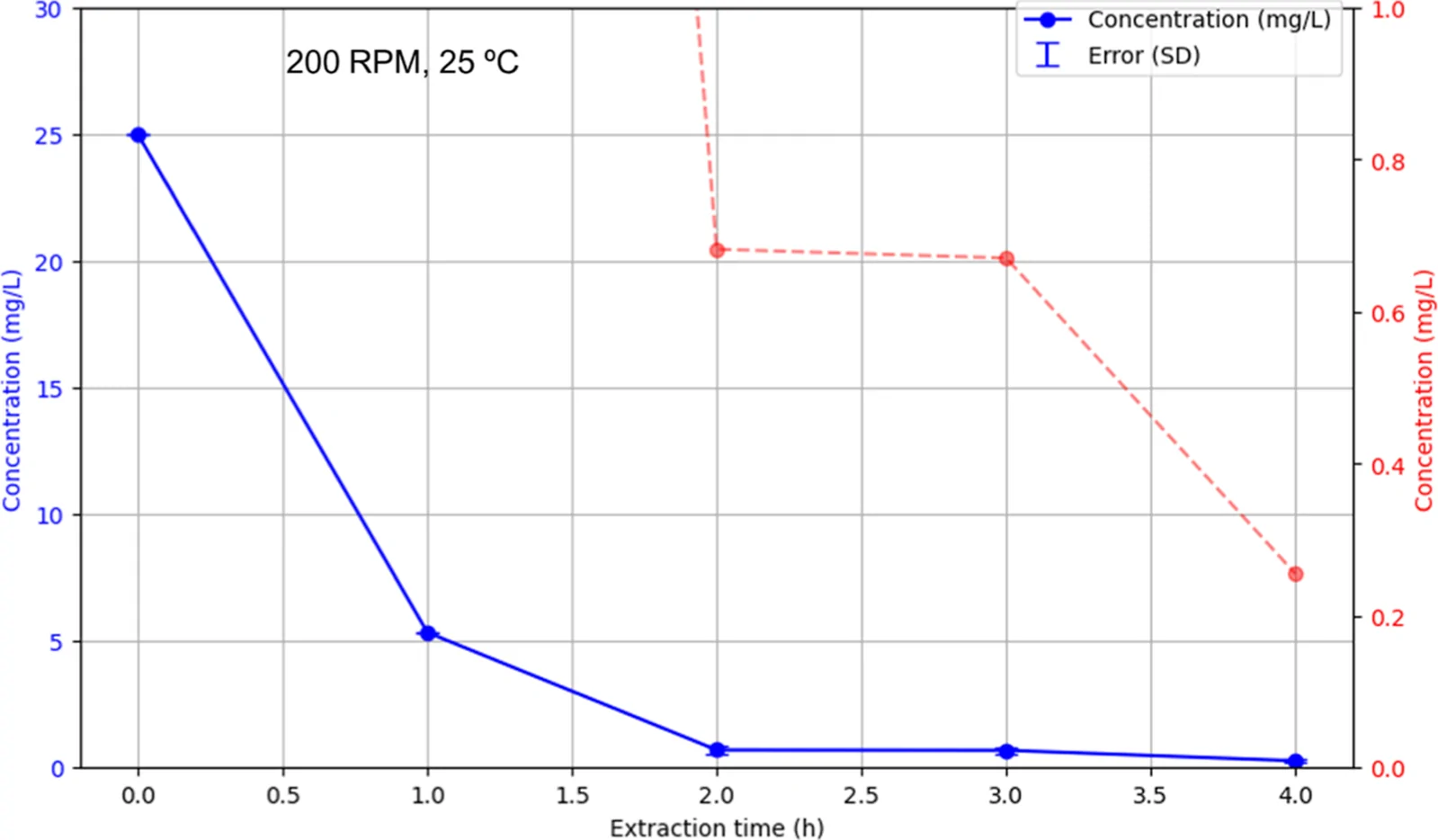
Extraction efficiency of Thy:Men (1:1) NADES for PS nanoplastics over time at 25 °C and pH 3.0.

The transfer of hydrophobic analytes into immiscible organic solvents depends on the affinity between the selected solvent and the polymer;^67^ Traditional organic solvents dissolve the plastic analytes completely, which prevents morphological characterization and quantification of particles. Alternative extraction methods, including cloud-point extraction (CPE) using surfactants or the use of green solvents such as NADES, have been developed to maintain particle integrity and minimize environmental impact.

From an ecological perspective, it is worth mentioning that, depending on the components and application, limited leaching of NADES components into water is considered acceptable, especially when the leached substances are much less harmful than the contaminants being removed.^20^ In any case, the retention of component quantities after interaction with water in a similar extraction system has already been observed, which highlights the efficiency of these systems in removing contaminants from water without the risk of cross-contamination.^20^

### 3.2 Molecular Dynamics Analysis

MD simulations were employed to elucidate the molecular-level mechanisms governing nanoplastic extraction by NADES, serving as a key tool to interpret system stability, interfacial behavior, and interaction energies. The Root Mean Square Deviation (RMSD) results were used to analyze the equilibration of the systems. Subsequently, the water–NADES interface was investigated through the density profiles of the substances in the simulation boxes. The IPE results were used to analyze the extraction capability of NADES toward PS nanoplastics in the simulated aqueous systems, and the phase transfer was evidenced by the number of contacts of PS during the production runs.

#### 3.2.1 RMSD and System Stability

The RMSD analysis was performed for the PS chains with the system used as reference to obtain the RMSD results for all NPT equilibration stages and NPT production simulations (Figure 2). The PS–water system reached equilibrium within the first 7 ns of equilibration (Figure 2A), and after 40 ns, it reached equilibrium in the production run. Figure 3 illustrates the time evolution of the system, showing the transition from initially dispersed PS chains (0 ns) to the formation of multiple small clusters (10–20 ns), followed by their coalescence into larger and more compact aggregates (30–50 ns). This behavior indicates a rapid aggregation process occurring within the first tens of nanoseconds, followed by stabilization marked by the formation of fewer and larger clusters. This observation is qualitatively consistent with the known tendency of nanoplastics to undergo homo- and heteroaggregation, as well as to interact with organic matter, microorganisms, and macromolecules in environmental systems.^68^

**Figure 2:**
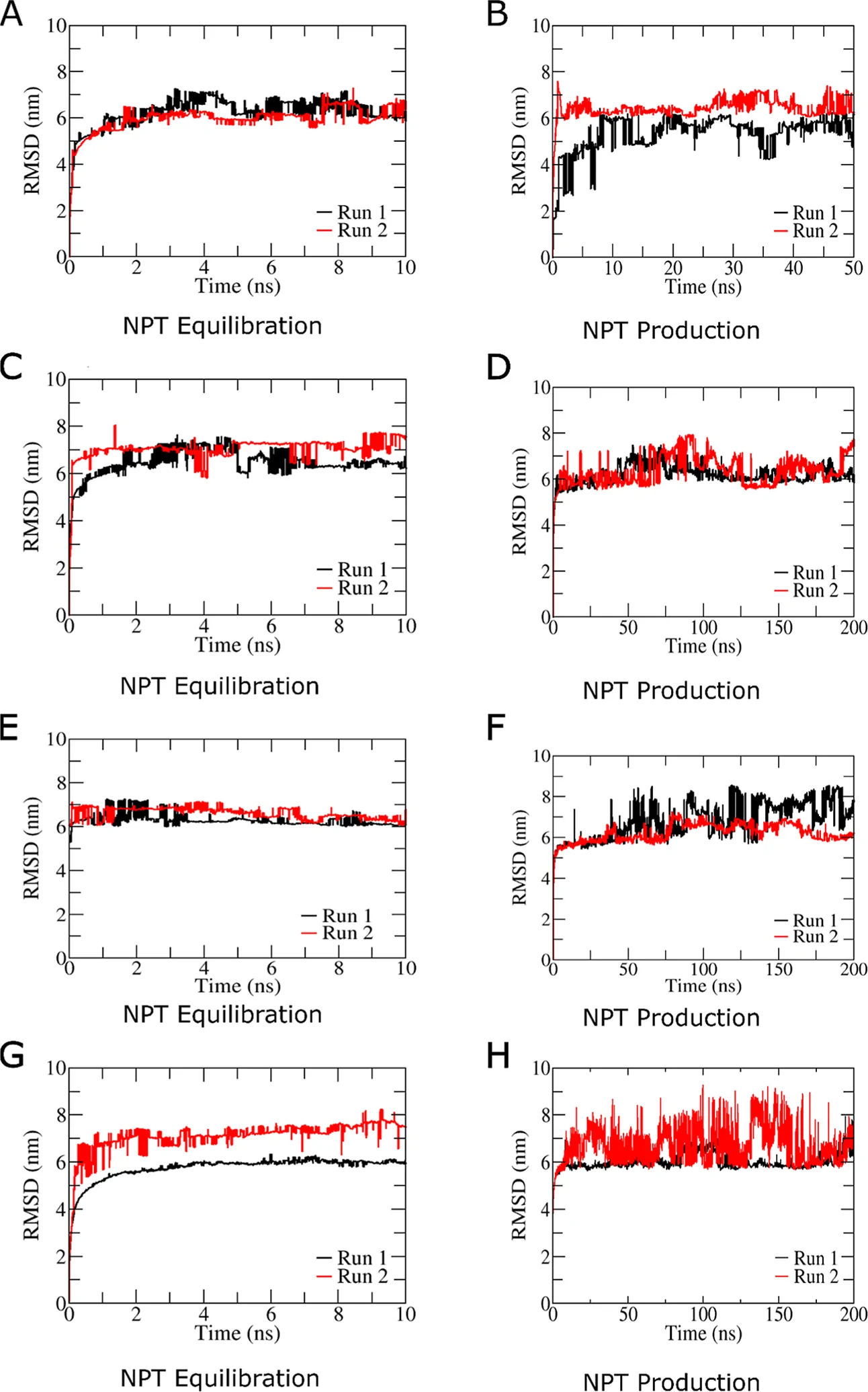
RMSD of (A) PS–water in NPT equilibration, (B) PS–water in NPT production, (C) PS–water–Thy:Men (1:1) in NPT equilibration, (D) PS–water–Thy:Men (1:1) in NPT production, (E) PS–water–Thy:Men (1:2) in NPT equilibration, (F) PS–water–Thy:Men (1:2) in NPT production, (G) PS–water–C_10_:C_12_ in NPT equilibration, and (H) PS–water–C_10_:C_12_ in NPT production. The black and red colors represent each replicate simulation.

**Figure 3:**
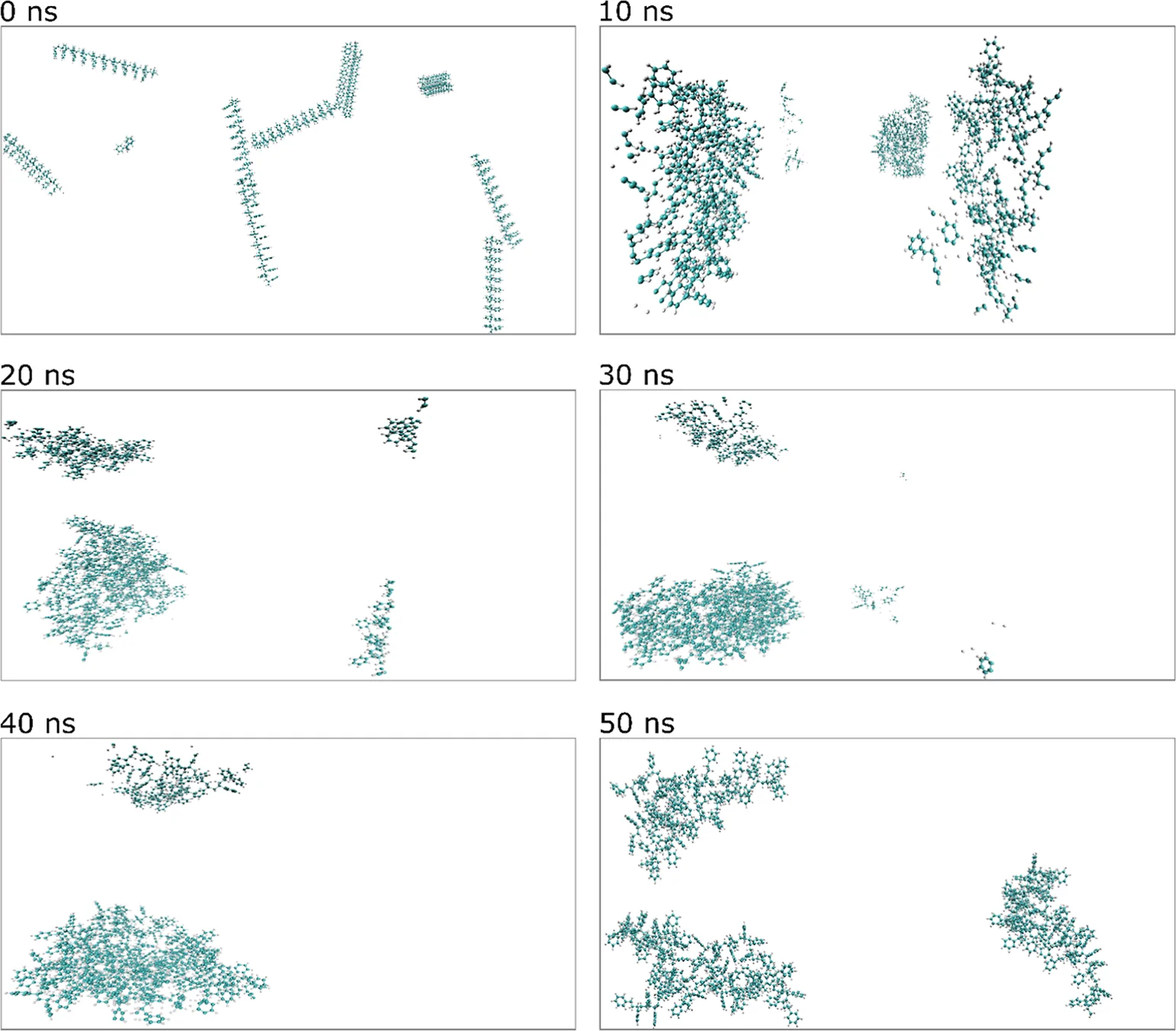
Snapshot of the PS–water system, in the initial random configurations and after all simulation steps.

Upon addition of the NADES components to the simulation boxes surrounding the initial system, the PS chains began migrating toward the NADES phase as phase separation progressed. For this reason, the simulations required longer times to stabilize, with around 7 ns for all equilibration runs, and around 150 ns for the production runs (Figure 2B,C, where the black and red lines represent each replicate of the systems simulated). The extended stabilization times reflect dynamic rearrangements at the water–NADES interface as the PS chains partition between the two phases.

#### 3.2.2 Interfacial Organization and Density Profiles

The immiscibility between NADES and water results in a well-defined interfacial layer that acts as a junction between them when they are mixed. This layer impacts the mass transfer between the two phases and the mobility of the PS chains between the phases. Figure 4 presents the number density profile along the *Z*-axis of the system components before the NPT equilibration and after the NPT production, accompanied by the snapshots of the systems.

**Figure 4:**
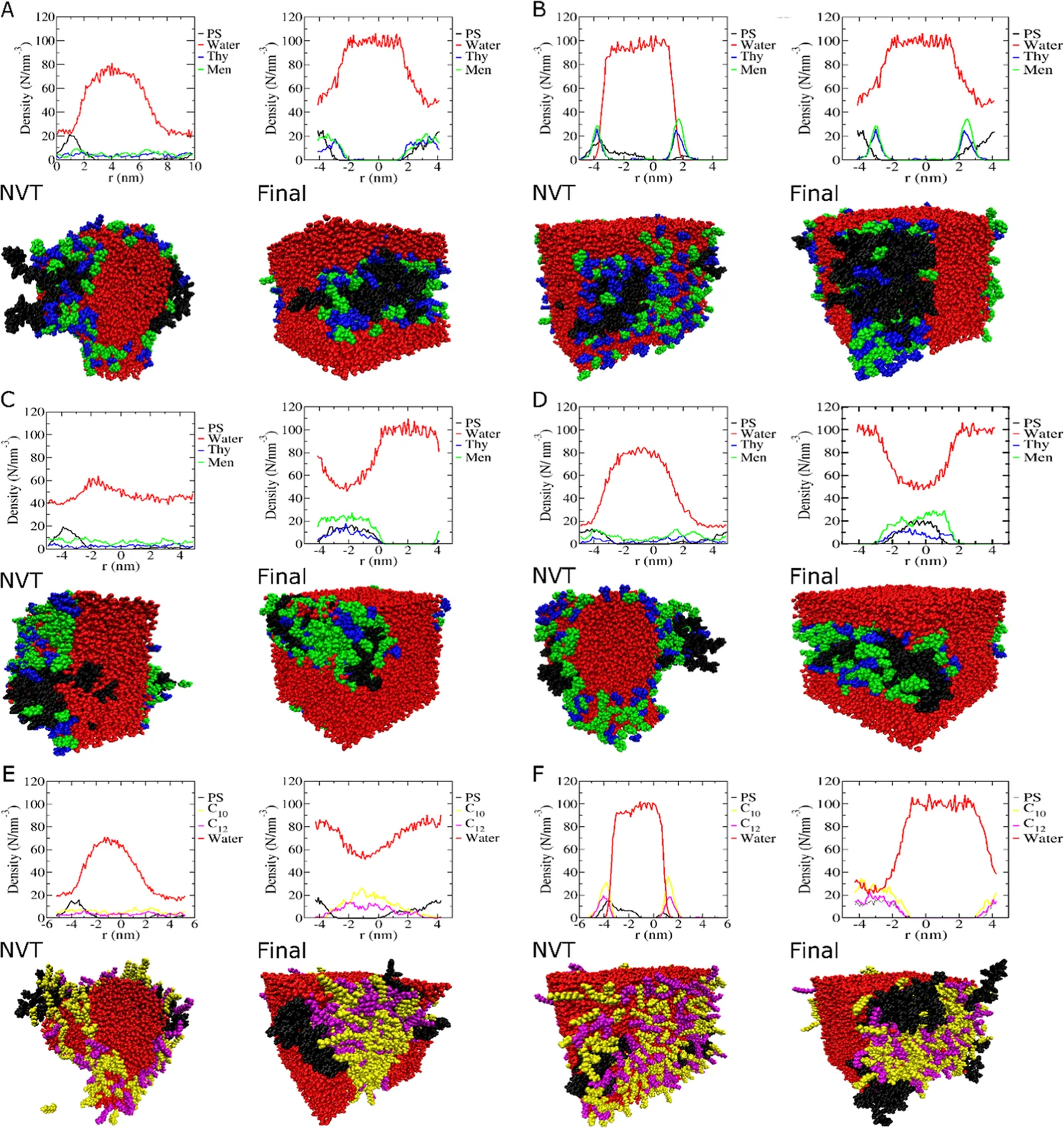
Density profile of the initial (after NVT) and final (after production) frame conformations of the extraction systems for (A) Thy:Men 1:1 run 1, (B) Thy:Men 1:1 run 2, (C) Thy:Men 1:2 run 1, (D) Thy:Men 1:2 run 2, (E) C_10_:C_12_, 1:2 run 1, and (F) C_10_:C_12_, 1:2 run 2. The representative snapshot of the system is presented above the plot, where red, black, blue, green, yellow, and magenta colors represent water, PS, Thy, Men, C_10_, and C_12_ respectively.

The simulation results show that the interface consists of both NADES components and water molecules, and that PS chains migrate from the aqueous phase toward the Thy:Men solvent. In contrast, the C_10_:C_12_ system exhibits phase segregation into distinct domains with incomplete separation. It is observed that Men tends to accumulate more at the interface compared to Thy for the Thy:Men NADES, as indicated by the larger Men peaks in the interfacial region. Meanwhile, C_10_ showed higher interfacial accumulation than C_12_ for the C_10_:C_12_ 2:1 system. Moreover, a higher ratio of Men in the NADESs leads to a greater proportion of Men within the interface.

The observations from MD analysis could potentially explain the differences in the extraction efficiencies between different NADESs. The acidic features of Men and its capacity to act as a hydrogen donor make it more accessible for hydrogen bond interactions with water.^69^ This chemical difference is expressed in the phase behavior, where menthol acts as a kind of bridge between PS and water, reducing the surface tension between these phases. This is also verified experimentally, with menthol being more soluble in water than thymol.^69,70^ These groups partially bind to water via hydrogen bonds and anchor to the PS via hydrophobic interactions with the aromatic ring and the chain. Thymol contributes primarily through π–π stacking interactions with the PS chains, but the excess menthol facilitates partial miscibility and organizes the interface.^6^

On the other hand, the aliphatic chain of the carboxylic acids on the C_10_:C_12_ mixture does not provide the π–π stacking and the components showed higher interaction with water, reducing the phase separation, which was observed by the formation of unstable emulsions. According to Ishtaweera, this can be explained by the poor affinity between PS and dodecanoic acid, although the authors obtained 71.7% removal efficiency for the same NADES with the same PS size.^20^ The more stable RMSD values and the well-segregated density profiles obtained for the Thy:Men NADES compared to C_10_:C_12_ 2:1 suggest a lower extraction efficiency of the eutectic mixture.

#### 3.2.3 Interaction Potential Energies

Table 3 summarizes the interaction potential energy (IPE) of all simulation components during production runs. The NPT analysis was included for discussion since it was observed that the phase separation started in the equilibration step, showing the PS affinity for the studied NADES.

**Table 3:** Average Interaction Potential Energies (IPE, kJ mol^–1^) Between PS and the Components of the Simulated Systems

NPT equilibration (0–7 ns)							NPT production (0–150 ns)						
systems/interactions	replicates	PS–water	PS–HBD	PS–HBA	water–HBD	water–HBA	systems/interactions	replicates	PS–water	PS–HBD	PS–HBA	water–HBD	water–HBA
PS–water	1	–5163.74 ± 412.76	–	–	–	–	PS–water (0–30 ns)	1	–4681.57 ± 135.39	–	–	–	–
	2	–5297.84 ± 313.44	–	–	–	–		2	–4704.37 ± 136.58	–	–	–	–
PS–water–Thy:Men (1:1)	1	–1898.25 ± 633.06	–1517.91 ± 259.92	–1479.29 ± 325.22	–3118.27 ± 274.79	–5336.83 ± 439.94	PS–water-Thy:Men (1:1)	1	–777.12 ± 146.35	–2039.78 ± 152.39	–2539.22 ± 277.99	–3498.57 ± 230.06	–4776.96 ± 379.87
	2	–2092.03 ± 677.53	–1569.52 ± 232.54	–1451.99 ± 357.16	–3857.10 ± 210.40	–5764.06 ± 570.98		2	–627.51 ± 192.37	–2223.87 ± 166.93	–2401.62 ± 171.72	–3535.60 ± 209.52	–4933.74 ± 294.53
PS–water–Thy:Men (1:2)	1	–2031.19 ± 503.50	–815.37 ± 126.78	–2354.34 ± 360.00	–2352.64 ± 308.70	–7480.39 ± 785.70	PS–water-Thy:Men (1:2)	1	–683.41 ± 231.79	–1376.73 ± 190.03	–3434.85 ± 301.73	–2480.34 ± 209.71	–6397.07 ± 308.30
	2	–1951.70 ± 422.35	–1016.39 ± 160.68	–2105.73 ± 203.50	–2189.66 ± 219.97	–6822.12 ± 651.33		2	–673.56 ± 188.92	–1334.17 ± 139.72	–3300.85 ± 241.01	–2532.30 ± 203.31	–6277.20 ± 382.43
PS–water–C_10_:C_12_ (2:1)	1	–2693.56 ± 251.92	–1398.13 ± 135.24	–656.27 ± 73.80	–4241.34 ± 269.67	–2112.63 ± 267.89	PS–water–C_10_:C_12_ (2:1)	1	–2683.62 ± 195.67	–1489.07 ± 139.94	–707.23 (±127.66)	–4025.52 ± 206.40	–2031.12 ± 165.07
	2	–2482.34 ± 226.01	–1324.21 ± 129.74	–1019.52 ± 125.06	–4650.37 ± 222.06	–2215.42 ± 182.52		2	–2476.72 ± 158.04	–1432.58 ± 152.82	–909.81 (±133.60)	–4485.88 ± 192.68	–2231.64 ± 155.46

NPT equilibration (0–7 ns)							NPT production (0–150 ns)						
systems/interactions	replicates	PS–water	PS–HBD	PS–HBA	water–HBD	water–HBA	systems/interactions	replicates	PS–water	PS-HBD	PS-HBA	water-HBD	water-HBA
PS–water	1	–4808.58 ± 127.28	–	–	–	–	PS–water (30–50 ns)	1	–4677.28 ± 117.05	–	–	–	–
	2	–4814.36 ± 127.24	–	–	–	–		2	–4497.65 ± 191.74	–	–	–	–
PS–water-Thy:Men (1:1)	1	–944.89 ± 71.59	–2073.16 ± 111.53	–1763.14 ± 114.32	–3368.33 ± 174.58	–5018.03 (±208.00)	PS–water–Thy:Men (1:1)	1	–596.58 ± 120.87	–2173.03 ± 122.48	–2487.18 ± 145.22	–3631.04 ± 242.67	–4708.21 ± 242.97
	2	–977.52 ± 106.62	–1907.61 ± 126.78	–2082.23 ± 118.65	–3608.89 ± 177.80	–5405.20 (±268.79)		2	–683.15 ± 184.84	–2323.52 ± 160.08	–2480.77 ± 185.54	–3411.04 ± 255.74	–4790.48 ± 246.87
PS–water-Thy:Men (1:2)	1	–1427.46 ± 86.41	–1030.88 ± 58.44	–2673.47 ± 102.59	–2107.86 ± 124.92	–6773.17 (±232.59)	PS–water–Thy:Men (1:2)	1	–716.45 ± 125.72	–1482.69 ± 152.98	–3728.55 ± 144.44	–2476.62 ± 191.06	–6073.54 ± 256.30
	2	–1431.66 ± 104.92	–1264.32 ± 76.52	–2387.28 ± 119.66	–2229.38 ± 130.99	–6457.20 (±248.55)		2	–498.78 ± 112.38	–1325.51 ± 101.87	–3344.60 ± 171.21	–2573.83 ± 182.88	–6071.83 ± 264.04
PS–water–C_10_:C_12_ (2:1)	1	–2545.47 ± 181.80	–1526.65 ± 101.35	–787.15 ± 98.03	–4097.52 ± 185.84	–2033.86 (±132.16)	PS–water–C_10_:C_12_ (2:1)	1	–2688.63 ± 121.81	–1309.10 ± 101.47	–732.87 ± 99.12	–3994.09 ± 203.37	–2051.26 ± 171.65
	2	–2357.80 ± 134.18	–1342.01 ± 111.34	–1013.62 ± 74.46	–4615.50 ± 207.12	–2288.72 (±143.44)		2	–2461.33 ± 119.18	–1398.16 ± 129.43	–823.30 ± 112.22	–4457.28 ± 192.75	–2260.48 ± 146.66

In the PS–water system, the average IPE interaction energy value was −4587.41 ± 112.30 kJ mol^–1^ for water interacting with the PS surfaces (Table 3). Upon addition of NADES components, the interactions changed, with values ranging between −498.78 kJ (Thy:Men 1:2) and −2688.63 kJ mol^–1^ (C_10_:C_12_ 2:1).

The periodic boundary conditions (PBC) used in the MD simulations account for the interactions between the PS/NADES molecules in the upper region and their replicated molecules on the lower surface. For the NADES Thy:Men 1:1, the water–Men IPE values were around 30% more negative than the water–Thy interaction, indicating stronger hydrogen bonding capability of menthol. Likewise, the PS-Men interactions were also the most favorable throughout the duration of the simulations, showing a more amphiphilic character compared to thymol.

The IPE values of the PS-Thy interaction were around 10% lower than the PS-Men interaction for the NADES Thy:Men 1:1, while the average number of contacts between PS-Thy was around 24% lower than that of PS-Men for the same NADES (Figure 5). This indicates that the PS-Men contribution has an individual lower energy contribution than PS-Thy but has a higher affinity.

**Figure 5:**
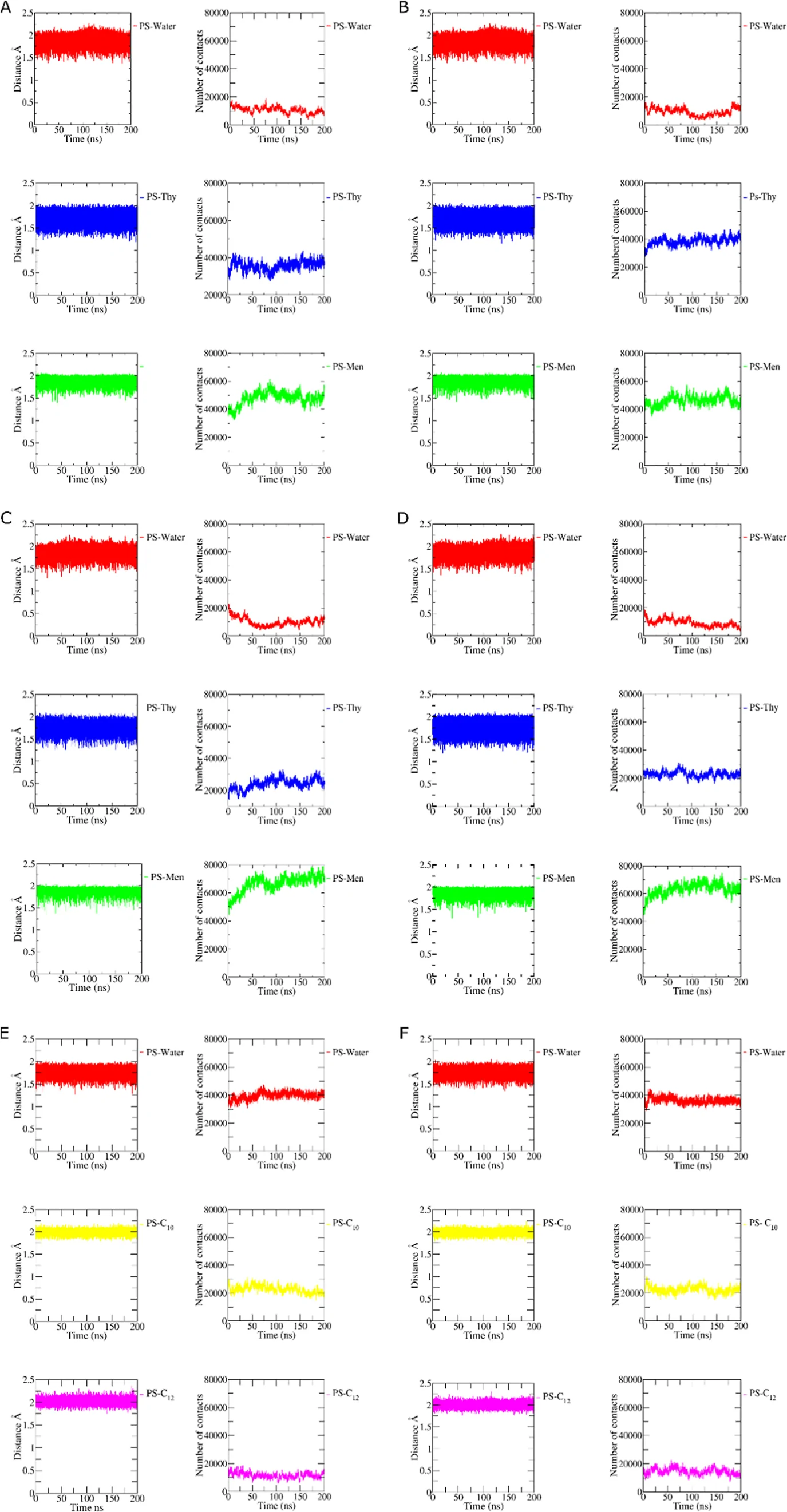
Minimal distance (Å) and number of contacts of PS with the other components of the extraction systems during the production simulations for (A) Thy:Men 1:1 run 1, (B) Thy:Men 1:1 run 2, (C) Thy:Men 1:2 run 1, (D) Thy:Men 1:2 run 2, (E) PS–water-C_10_:C_12_ 2:1 run 1, and (F) PS–water-C_10_:C_12_ 2:1 run 2. The red, blue, green, yellow, and magenta colors represent the interaction of PS with water, Thy, and Men, C_10_ and C_12_, respectively.

For the C_10_:C_12_ 2:1 eutectic mixture, the component contributions were proportionally close for interactions with PS and water, but the number of contacts was numerically close, but not proportionally consistent. It is also worth mentioning that the IPE value of PS–water and PS-NADES for this solvent were very similar, indicating the lower extraction efficiency for the proposed system.

The interaction energy of the NADES components with the nanoplastics was almost completely governed by Lennard-Jones (LJ) interactions, suggesting that extraction is mediated due to van der Waals forces, given that the PS surface has no significant polarity.^71^ The electrostatic contribution is slightly lower than the LJ contribution to the PS-water interaction energies (e.g., −300.82 vs −415.62 kJ/mol for the PS + water + Thy:Men 1:2 after RMSD stabilization).

While the PS-Thy and PS-Men, PS-C_10_ and PS-C_12_ are almost completely governed by LJ interactions (e.g., 15.63 electrostatic vs −1498.32 kJ/mol LJ for PS-Thy; −163.66 electrostatic vs −3892.21 kJ/mol LJ for PS-Men in the PS + water + Thy:Men 1:2 system; −43.27 vs 1310.36 for PS-C_10_; −16.1835 vs −775.00 for PS-C_12_ in the PS + water + C_10_:C_12_ 2:1 system), the water–Thy interaction shows a more balanced contribution between electrostatic and LJ terms, whereas the water–Men interaction shows a stronger electrostatic contribution (e.g., 1132.36 electrostatic vs 1344.26 kJ/mol LJ for water–Thy; −4040.85 electrostatic vs 2032.69 kJ/mol LJ for water–Men in the PS + water + Thy:Men 1:2 system). This indicates that the hydroxyl group of menthol is more available for hydrogen bonding with water.

This behavior was not the same as that observed by Garmroodi et al.^69^ In the article in question, the authors observed a similar predominance of electrostatic energy over LJ for both water-thy (approximately 70/30%) and water-men interactions (approximately 75/25%). Although this trend was also observed for water–Men in the present work, the relative contribution was significantly different for water-thy (approximately 46/54%) and closer for water-men and (67/33%). This can be explained of differences in the density profiles of the systems. In the referenced study, thymol and menthol were more uniformly distributed throughout the aqueous simulation box, leading to a relatively homogeneous interaction region. In contrast, in the present work, thymol shows a preferential accumulation at the surface of the PS interface, which shifts the spatial distribution of intermolecular contacts. As a result, the interaction pattern becomes more interfacially dominated, with a reduced contribution from bulk-phase water–NADES interactions.

In another study of systems composed by Thy:Men + water, Deepika et al.^72^ also reported higher water solubility for thymol-based NADESs. While, in the present study, simulations indicate greater interfacial activity of menthol, corroborating experimental extraction trends. Furthermore, the authors observed greater water solubility of Thy:Men NADES as the mass fraction of thymol in the mixture increased.

For the C_10_–water and C_12_–Water interactions, the electrostatic interactions predominated, with proportionally similar contribution values from the NADES components (−2651.07 electrostatic vs −1540.77 kJ/mol) potential for water-C_10_; 1333.83 electrostatic vs −843.00 kJ/mol potential for water-C_12_. Finally, based on average IPE results, it can be concluded that Thy:Men 1:2 showed slightly more negative total interactions (−4940.68 kJ/mol) than Thy:Men 1:1 (−4732.25 kJ/mol) which is consistent with the similar extraction efficiencies observed experimentally. However, these two NADES showed much stronger affinity than C_10_:C_12_ 2:1 (−2041.97 kJ/mol), suggesting their superior overall affinity toward PS nanoplastics and, consequently, higher extraction potential.

### 3.3 Mechanistic Interpretation of Nanoplastic Extraction by NADES

The experimental and molecular dynamics results provide a consistent picture of how Thy:Men NADES promote the extraction of hydrophobic nanoplastics from aqueous media. The overall mechanism can be described as a sequence of interfacial adsorption, partial desolvation, and stabilization of the polymer within the NADES phase (Figure 6).

**Figure 6:**
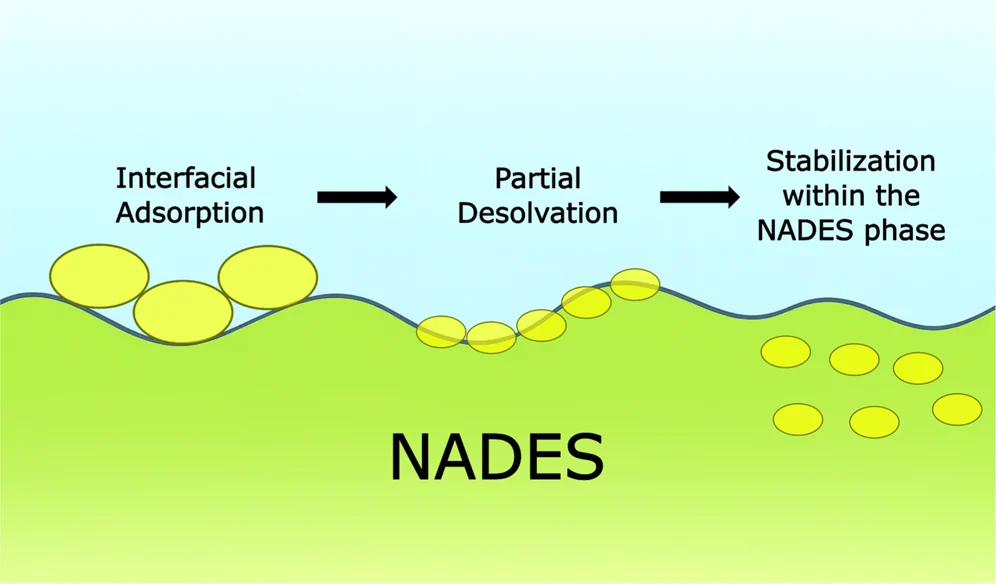
PS nanoplastics extraction from contaminated waters using Thy:Men NADESs: Initially, thorough mixing emulsifies the NADES and the aqueous phase, promoting interfacial adsorption. The next step is the partial desolvation of nanoparticles. Finally, the particles are captured and retained in the NADES phase.

In aqueous systems, PS nanoparticles exhibit limited stability due to their strong hydrophobicity, which promotes aggregation (Figures 3 and 4). Upon addition of the NADES phase, small hydrophobic droplets are formed and dispersed throughout the aqueous medium. The large surface area of these droplets enables multiple transient contacts between Thy and Men molecules and the PS surface, allowing physical adsorption driven by van der Waals dispersion and π–π interactions.^6,64^ The hydrogen-bonding ability of the Thy:Men NADES further modulates this process by influencing the interfacial tension and the polarity gradient at the water–NADES boundary.^20^

The molecular dynamics simulations revealed that menthol molecules accumulate at the interface, forming a flexible amphiphilic layer capable of interacting simultaneously with both water and PS. The hydroxyl group of menthol forms transient hydrogen bonds with water molecules, while its hydrophobic isopropyl group interacts with the aromatic PS backbone through dispersion forces.^6,20^ This dual behavior effectively acts as a molecular bridge, reducing the interfacial energy barrier and facilitating the migration of PS from the aqueous to the NADES phase.

In contrast, thymol plays a more specialized role in the subsequent stabilization of PS within the NADES. Its aromatic ring establishes π–π stacking interactions with the phenyl groups of PS, while the hydroxyl group provides occasional hydrogen bonding with surrounding menthol molecules.^69^ This combination of specific (π–π) and nonspecific (LJ) interactions explains the high affinity and stability of the Thy:Men mixtures, particularly the 1:1 composition, which balances polarity and hydrophobicity. The presence of excess menthol (Thy:Men 1:2) enhances phase transfer through increased interfacial fluidity but may slightly reduce polymer retention due to the weaker π–π contribution, aligning with experimental results.^64^

The extraction process can thus be understood as a thermodynamically favorable phase transfer, where the PS chains migrate to minimize the system’s free energy. The dominance of LJ interactions over electrostatic contributions in the IPE analysis confirms that dispersion and hydrophobic forces are the main driving factors. The interfacial organization of NADES molecules, particularly the accumulation of menthol and partial penetration of thymol, creates a gradient of polarity that promotes the entrapment and stabilization of PS within the organic phase.

Overall, these findings suggest that Thy:Men NADES-mediated extraction of nanoplastics follows a “soft adsorption-migration-stabilization” mechanism, combining interfacial phenomena with molecular recognition effects. This mechanism accounts for the high extraction efficiencies observed experimentally (up to 97%) and explains the superior performance of thymol-based NADES compared with other hydrophobic systems reported in the literature.^6,19,20,64,67^

From an environmental perspective, the ability of NADES to extract nanoplastics through mild, nondestructive physical interactions represents an attractive alternative to conventional organic solvents. These green solvents enable particle retention and recovery without full dissolution, minimizing analytical interference and potential ecological risks.^6,20,64^

## 4 Conclusions

This study validates the potential of menthol-based NADES to extract nanoplastics from aqueous environments. The solvent composition, pH and the specific interactions between the solvents and nanoplastics were the main factors impacting extraction efficiency. The Lennard-Jones (LJ) interactions between the NADES components (high a higher contribution from menthol) and nanoplastics were the main driving forces for the extraction.

Among the tested systems, Thy:Men 1:1 showed the highest extraction efficiencies at pH 3.0, 7.0, and 9.0 (97.14 ± 0.49%, 75.35 ± 20.90%, and 78.19 ± 1.36%, respectively), with the lowest standard deviations, while C_10_:Men 1:2 showed the highest efficiency at pH 4.0 (74.17 ± 19.24). The high standard deviation values may be mainly related to mass transfer problems and phase separation, as reported in other cases in the literature, except at more acidic pH values, where extraction was favored. Under acidic conditions (pH 3.0), extraction was favored, reaching equilibrium after approximately 2 h for the Thy:Men (1:1) system.

The Thy:Men 1:2 solvent showed slightly more stable interaction potential energy (IPE) values in simulations, however the stronger hydrogen-bonding affinity of menthol with water likely hindered complete phase separation, explaining the slightly lower experimental efficiencies.

The combined use of experimental and MD simulations provided complementary insights, with MD elucidating the molecular-level mechanisms underlying solvent–polymer interactions and extraction behavior. These findings contribute to the rational design and optimization of solvent systems for nanoplastic removal. Although the potential has been demonstrated, further studies, including detailed mass transfer investigations, are necessary to support the development of more precise and effective strategies for removing nanoplastics from aqueous environments.

## Supplementary Material



## Data Availability

The data sets generated and analyzed during the current study are included in the manuscript and its Supporting Information Additional data are available from the corresponding author on reasonable request.

## Notes

Calibration curves for polystyrene microspheres in water, two- and three-dimensional structures of the molecular systems used for the DFT optimizations and molecular dynamics simulations, and DFT-optimized geometries and energies (PDF)
