# Supplementary material for: Efficient Extraction of Nanoplastics from Aqueous Media Using Natural Deep Eutectic Solvents: Experimental and Molecular Insights
Source: ACS Omega. 2026 Aug 18;11(34):50908–21. doi: 10.1021/acsomega.6c02548 (PMC13624943; doi:10.1021/acsomega.6c02548)
Supplement: Supplementary file 1 [file ao6c02548_si_001.pdf]

Supporting information

Efficient Extraction of Nanoplastics from Aqueous Media Using Natural Deep Eutectic Solvents (NADES): Experimental and Molecular Insights

Francisco Thálysson Tavares Cavalcante[1], Lucas Lima Bezerra[2], Norberto de Kássio Vieira Monteiro[2], and Rilvia Saraiva de Santiago-Aguiar[1]

[1]Department of Chemical Engineering, Center for Technology, Federal University of Ceara, Campus do Pici, bl. 709, 60455-760, Fortaleza, Ceará, Brazil.

[2]Department of Analytical and Physical Chemistry, Center of Sciences, Federal University of Ceara, Campus do Pici, bl. 940, 60440-900, Fortaleza, Ceará, Brasil.

* Corresponding author: rilvia@ufc.br

Summary:

Number of figures: 1

(A)

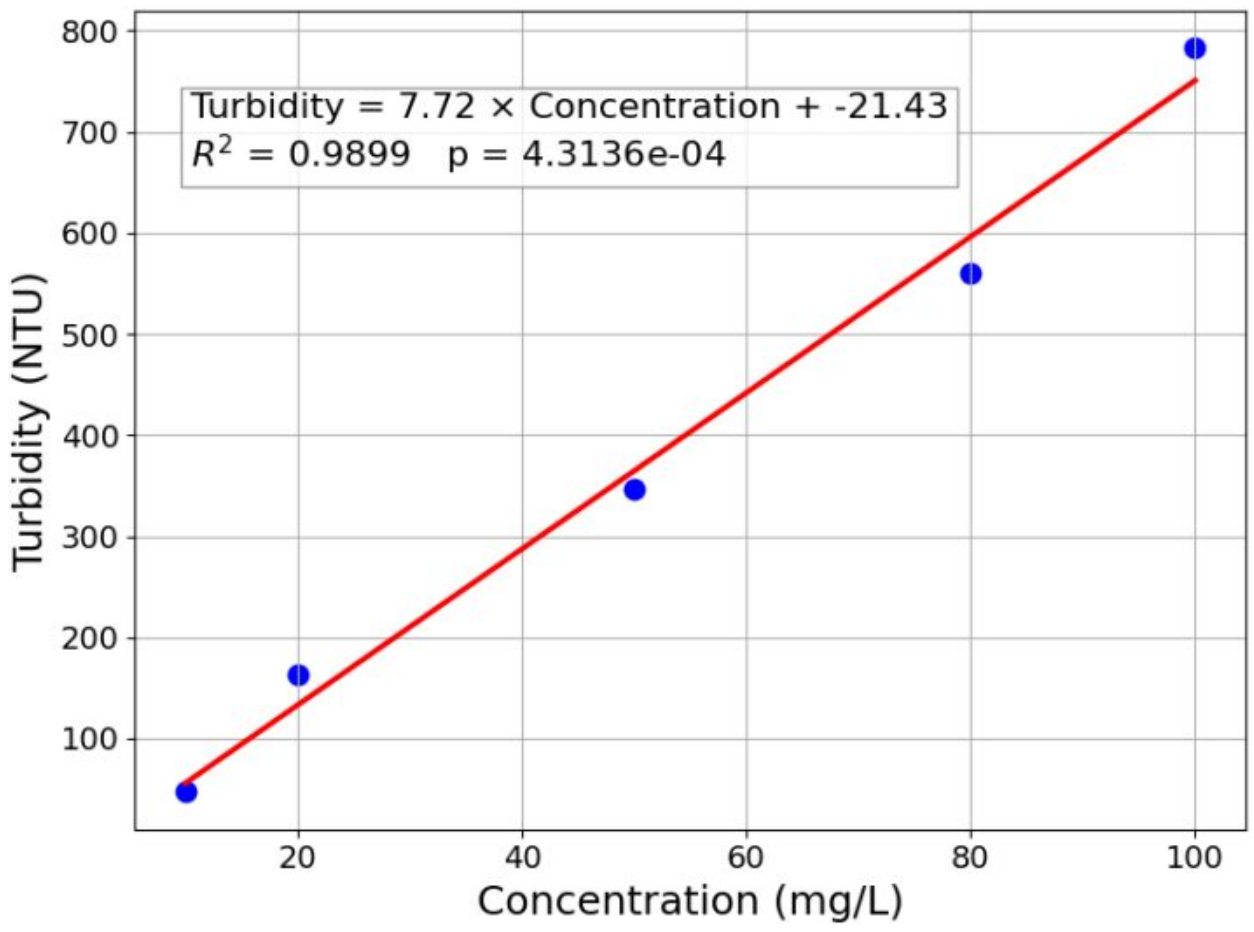

(B)

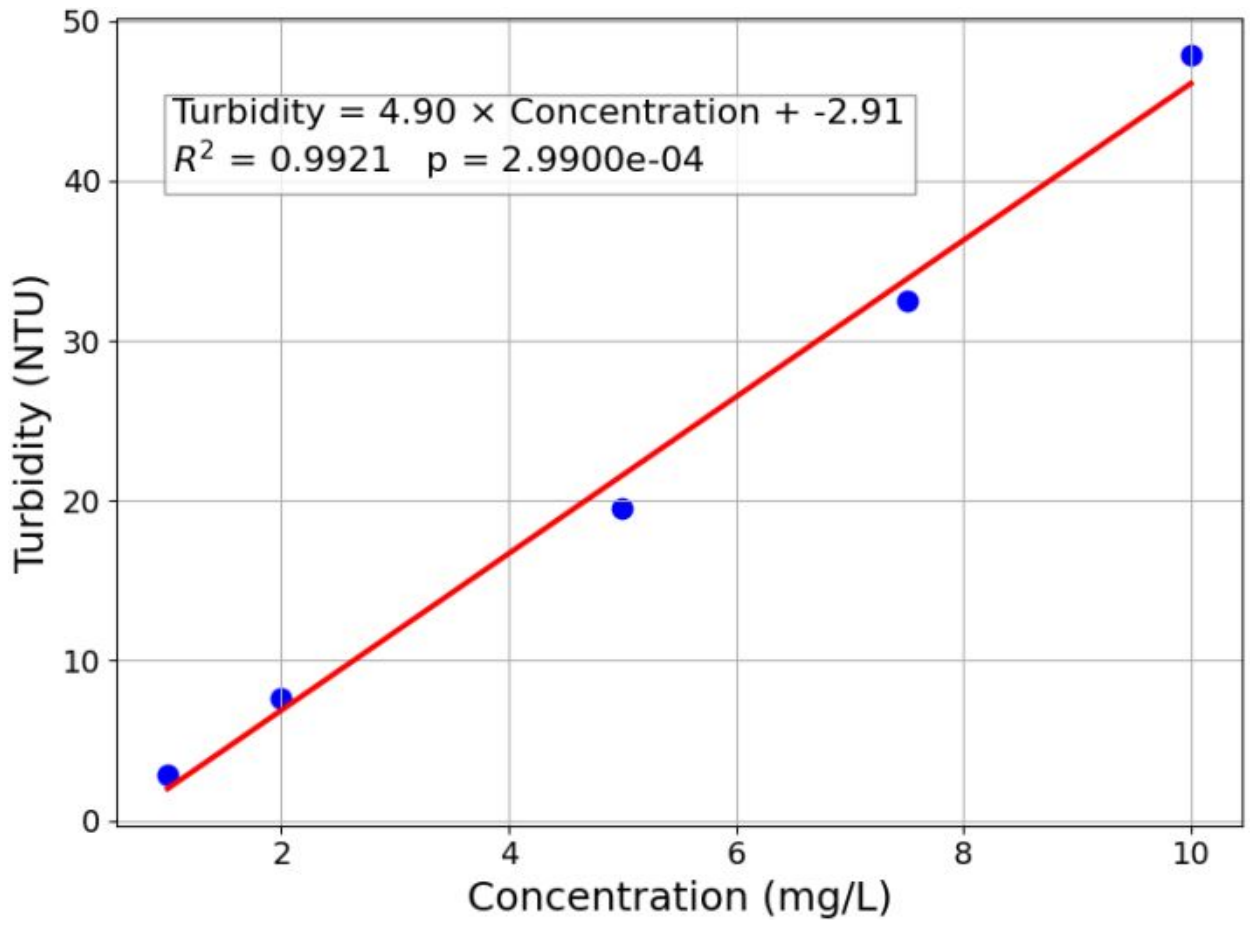

Figure S1. Calibration curves of turbidity (NTU) versus concentration (mg/L) for 500 nm PS microspheres in water: (A) 1–10 mg/L and (B) 10–100 mg/L.

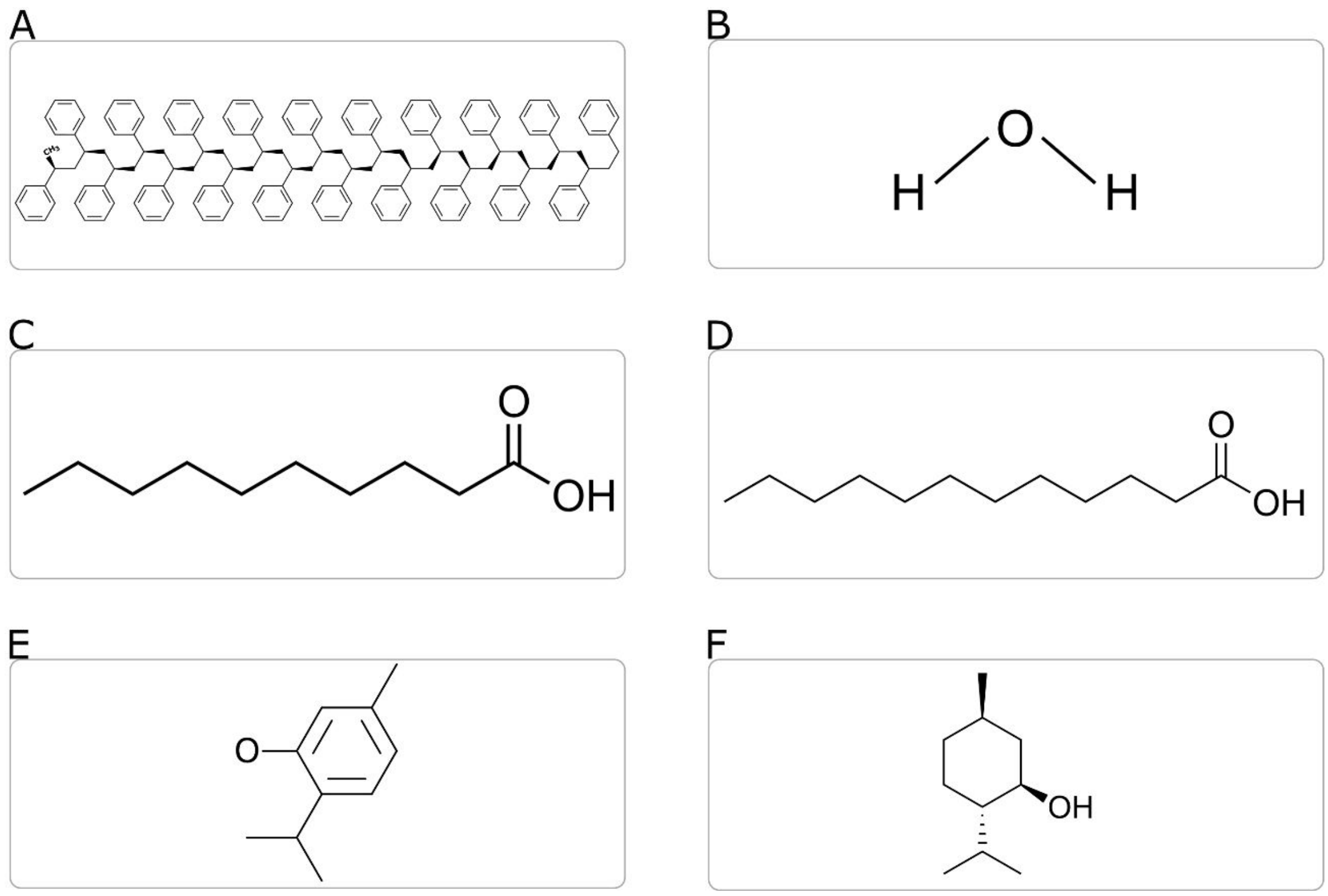

Figure S2. Two-dimensional structures of polystyrene (A), water (B), decanoic acid (C), dodecanoic acid (D), thymol (E), and menthol (F) used as input for the optimizations.

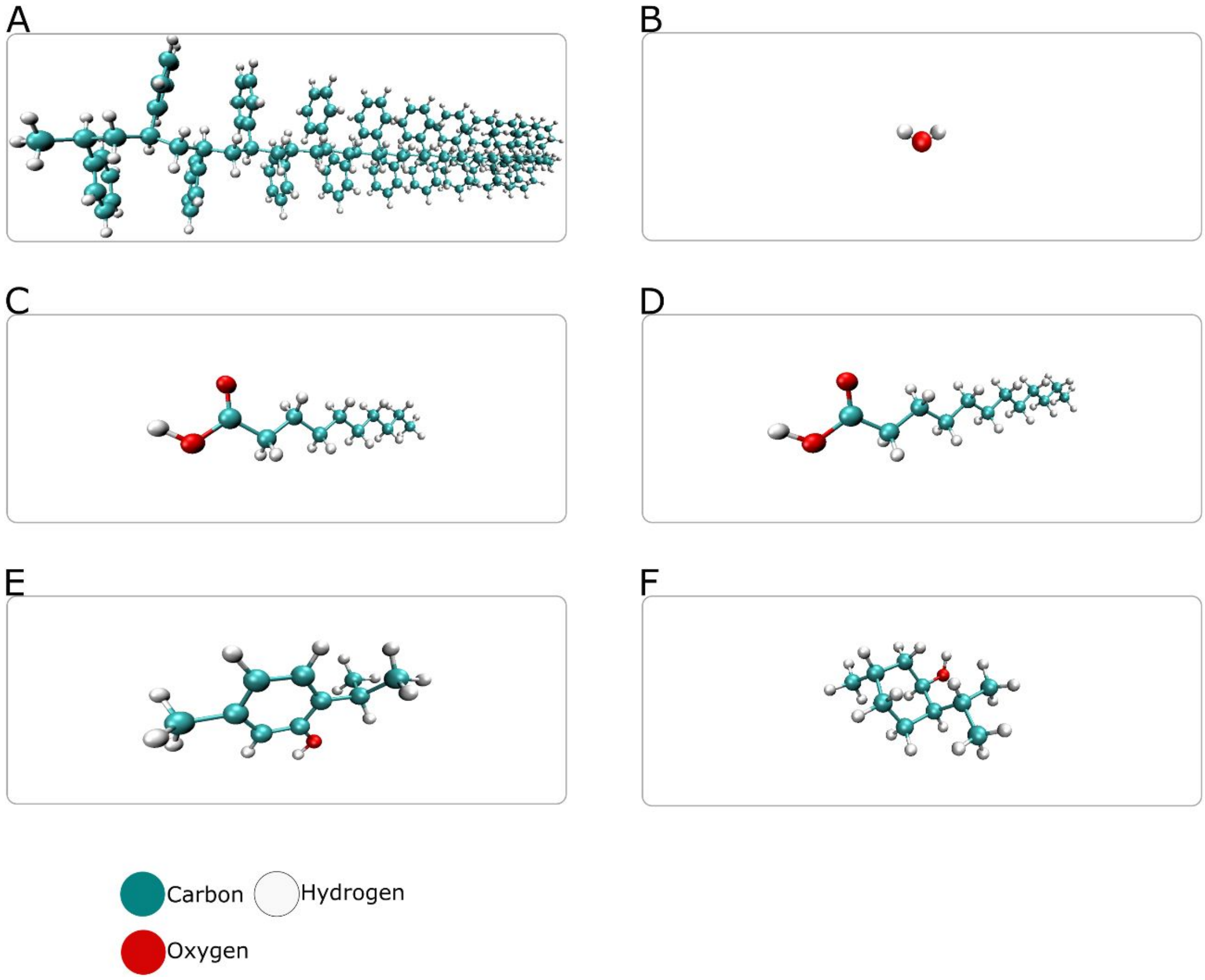

Figure S3. Three-dimensional structures of polystyrene (A), water (B), decanoic acid (C), dodecanoic acid (D), thymol (E), and menthol (F) used as input for the MD simulations.

Text S1: DFT-Optimized Geometries and Energies

Name: Decanoic acid

Method: B3LYP/6-311g(d,p)

Charge = 0 Multiplicity = 1

Atom and Cartesian coordinates (Å)

Atom	X	Y	Z
O	-5.877383	-0.705710	-0.003009
O	-4.931254	1.330418	0.003531
C	1.600078	-0.439108	0.000654
C	0.286473	0.350821	-0.001816
C	2.849776	0.448665	-0.001798
C	-0.962992	-0.536962	0.000863
C	4.163983	-0.340191	0.001122
C	-2.271289	0.260589	-0.001575
C	5.414286	0.546793	-0.001710
C	-3.508695	-0.635856	0.001414
C	6.722210	-0.249736	0.001623
C	-4.809882	0.132218	0.000738
H	1.625528	-1.101610	-0.874196
H	1.625282	-1.096432	0.879410
H	0.260205	1.007932	-0.880345
H	0.260126	1.013195	0.872747
H	2.824072	1.111398	0.872806
H	2.824562	1.105889	-0.880571
H	-0.937388	-1.199685	-0.873982
H	-0.937402	-1.194232	0.879819
H	4.190257	-0.997371	0.880174

H	4.190551	-1.003452	-0.873337
H	-2.305971	0.917188	-0.876773
H	-2.305966	0.922778	0.869350
H	5.388564	1.202863	-0.880243
H	5.387984	1.209278	0.871973
H	-3.519906	-1.302808	-0.868142
H	-3.518759	-1.297728	0.874970
H	6.792900	-0.890081	0.886501
H	7.593305	0.411216	0.000116
H	6.794050	-0.895633	-0.879112
H	-6.666584	-0.143772	-0.002432

Total electronic energy
−543.751525749 Hartree

Geometry optimization
Converged to a stationary point.

Vibrational analysis
The structure corresponds to a true minimum (0 imaginary frequencies).

Name: Dodecanoic acid

Method: B3LYP/6-311g(d,p)

Charge = 0 Multiplicity = 1

Atom and Cartesian coordinates (Å)

Atom	X	Y	Z
O	7.140333	-0.675935	-0.000683
O	6.175938	1.351702	-0.000278
C	-1.597203	0.397512	0.000036
C	-0.339130	-0.478345	0.000031
C	-2.903742	-0.404357	-0.000055

C	0.966967	0.323965	0.000353
C	-4.162073	0.471191	-0.000059
C	2.224681	-0.552134	0.000144
C	-5.468650	-0.330266	-0.000100
C	3.525528	0.257577	0.000514
C	-6.727660	0.544397	-0.000041
C	4.771213	-0.627395	0.000538
C	-8.027687	-0.264999	-0.000200
C	6.065263	0.152528	-0.000179
H	-1.578043	1.057739	-0.876603
H	-1.578109	1.057650	0.876744
H	-0.358313	-1.138657	0.876712
H	-0.358095	-1.138329	-0.876902
H	-2.922729	-1.064624	0.876649
H	-2.922663	-1.064541	-0.876823
H	0.987029	0.983658	0.877112
H	0.987133	0.984222	-0.875979
H	-4.143126	1.131415	0.876677
H	-4.143089	1.131468	-0.876752
H	2.205305	-1.212041	-0.876985
H	2.205167	-1.212667	0.876799
H	-5.488628	-0.990697	-0.876871
H	-5.488629	-0.990775	0.876610
H	3.553874	0.917083	0.873732
H	3.554195	0.917476	-0.872388
H	-6.708180	1.203798	0.876165
H	-6.708111	1.204004	-0.876091
H	4.788233	-1.291152	0.872464
H	4.787682	-1.292039	-0.870713
H	-8.092784	-0.908224	-0.883417
H	-8.092227	-0.909421	0.882190
H	-8.905366	0.387213	0.000521
H	7.924257	-0.106733	-0.001057

Total electronic energy
-622.399952829 Hartree

Geometry optimization
Converged to a stationary point.

Vibrational analysis
The structure corresponds to a true minimum (0 imaginary frequencies).

Name: Thymol

Method: B3LYP/6-311g(d,p)

Charge = 0 Multiplicity = 1

Atom and Cartesian coordinates (Å)

Atom	X	Y	Z
O	-0.338210	2.277951	0.369218
C	-2.098891	-0.045188	0.196356
C	-0.578477	-0.136941	0.131241
C	-2.784061	-1.340619	0.652004
C	-2.684284	0.432429	-1.150229
C	0.206881	1.029321	0.208986
C	0.101188	-1.343826	-0.046392
C	2.257112	-0.240778	-0.065483
C	1.595057	0.973779	0.115233
C	1.489122	-1.403799	-0.146326
C	3.763927	-0.284836	-0.149479
H	-2.355769	0.708568	0.957876
H	-2.677525	-2.133798	-0.093187
H	-3.854622	-1.169213	0.791867
H	-2.371067	-1.703011	1.596332
H	-2.238912	1.376643	-1.473360
H	-3.767330	0.571858	-1.079627
H	-2.485721	-0.307705	-1.930180
H	-0.463763	-2.266188	-0.109473

H	2.146017	1.905405	0.186303
H	1.975489	-2.363129	-0.286591
H	4.145133	0.459195	-0.854546
H	4.219551	-0.072586	0.823431
H	4.114506	-1.266993	-0.472764
H	-1.285759	2.200101	0.514720

Total electronic energy
-464.852985369 Hartree

Geometry optimization
Converged to a stationary point.

Vibrational analysis
The structure corresponds to a true minimum (0 imaginary frequencies).

Name: Menthol

Method: B3LYP/6-311g(d,p)

Charge = 0 Multiplicity = 1

Atom and Cartesian coordinates (Å)

Atom	X	Y	Z
O	-0.414950	2.392700	-0.269522
C	-0.680042	-0.063295	-0.348951
C	0.112196	1.144774	0.193116
C	-0.047887	-1.372273	0.165156
C	2.240087	-0.274823	0.359124
C	1.605542	1.034788	-0.135205
C	1.443744	-1.473646	-0.178975
C	-2.206811	0.057132	-0.096954
C	3.725400	-0.358244	-0.005720

C	-3.006678	-0.915340	-0.976923
C	-2.610206	-0.092643	1.378032
H	-0.545525	-0.046787	-1.443360
H	-0.005590	1.193126	1.280741
H	-0.581261	-2.233383	-0.247703
H	-0.162643	-1.431386	1.253929
H	2.160219	-0.289649	1.455650
H	2.122401	1.901675	0.288424
H	1.726231	1.098474	-1.227641
H	1.857113	-2.405621	0.222241
H	1.566780	-1.523212	-1.269471
H	-2.474363	1.073569	-0.402683
H	4.284608	0.486255	0.407616
H	4.176138	-1.278223	0.378232
H	3.862211	-0.348900	-1.092357
H	-2.816348	-1.959801	-0.710375
H	-4.081166	-0.742751	-0.865242
H	-2.759233	-0.791088	-2.035847
H	-2.427090	-1.105394	1.749336
H	-3.678325	0.109808	1.498498
H	-2.073450	0.607014	2.023679
H	-0.283174	2.426095	-1.223695

Name: Water

Method: B3LYP/6-311g(d,p)

Charge = 0 Multiplicity = 1

Atom and Cartesian coordinates (Å)

Atom	X	Y	Z
O	0.000000	0.000000	0.117025
H	0.000000	0.763627	-0.468098

H -0.000000 -0.763627 -0.468098

Total electronic energy
-76.4584627353 Hartree

Geometry optimization
Converged to a stationary point.

Vibrational analysis
The structure corresponds to a true minimum (0 imaginary frequencies).

Name: Polystyrene

Method: PM7
Charge: 0
Multiplicity: Singlet
Solvation model: COSMO (ε = 78.4)

Atom and Cartesian coordinates (Å)

Atom	X	Y	Z
C	-8.087090662	0.254226541	-0.021784471
H	-7.678198906	-0.773911854	-0.011145493
H	-7.711159247	0.720359503	-0.951597096
C	-7.585211803	1.026677239	1.208448515
H	-7.955202505	0.488238922	2.123470753
C	-6.048348075	1.012998758	1.239450057
H	-5.642044569	1.538706070	0.354788167
H	-5.679468105	-0.027363376	1.169943496
C	-5.535117090	1.681167082	2.524544392
H	-5.898237306	2.745315445	2.523251152

C	-3.998150910	1.704329277	2.523478251
H	-3.628358784	2.160642607	1.586386872
H	-3.598373489	0.672895794	2.537269533
C	-3.478067031	2.481832645	3.743047391
H	-3.841648818	1.950463511	4.664905192
C	-1.941332278	2.461903147	3.759269565
H	-1.540665899	2.988865812	2.872917024
H	-1.577430639	1.420250953	3.683147614
C	-1.413887168	3.123468915	5.042502611
H	-1.764732824	4.191708422	5.042502723
C	0.123174306	3.128837500	5.035411040
H	0.493946356	3.580106662	4.096528867
H	0.511647290	2.092634386	5.048186339
C	0.658235603	3.901052223	6.251827262
H	0.303808295	3.369013467	7.176515876
C	2.195572622	3.879627643	6.253136303
H	2.588104107	4.386683793	5.351725054
H	2.558216889	2.836398775	6.196973188
C	2.733455024	4.570172576	7.516137169
H	2.364089053	5.632379018	7.506576084
C	4.270348467	4.606738418	7.485525519
H	4.618817306	5.072437175	6.545316650
H	4.680006473	3.579362456	7.486817846
C	4.801604917	5.382379598	8.701966645
H	4.446828909	4.842898613	9.623559475
C	6.338809876	5.370704575	8.711160651
H	6.734747358	5.928831799	7.842541223
H	6.707736872	4.333948983	8.600614193
C	-9.623608317	0.215898606	-0.039564228
H	-9.997539688	1.276102957	-0.047185017
C	-10.112175184	-0.459742751	-1.331201211
H	-9.721566974	-1.492061216	-1.399928424
H	-9.708082942	0.076573821	-2.210484632
C	-11.647836443	-0.476968148	-1.376582313
H	-12.016289134	-1.021520965	-0.464418430
C	-12.131828644	-1.260224334	-2.609096307
H	-11.735116468	-2.291998858	-2.579635334
H	-11.734747897	-0.808503888	-3.536977565
C	-13.667106700	-1.273964507	-2.646397253
H	-14.018494239	-0.204496959	-2.641516612
C	-14.178932045	-1.915711163	-3.947390240

H	-13.828848429	-2.960557380	-4.031972750
H	-13.763389474	-1.383359423	-4.822150678
C	-15.715491196	-1.869632702	-3.962568098
H	-16.075568260	-2.432392824	-3.055865865
C	-16.283042102	-2.582326053	-5.200361664
H	-15.853130389	-3.597212060	-5.284569432
H	-15.993945917	-2.050215229	-6.124658525
C	-17.814829395	-2.652333005	-5.086044334
H	-18.197521849	-1.596067209	-5.030202712
C	-18.436093583	-3.306707862	-6.331933108
H	-18.134202739	-4.367419030	-6.403602659
H	-18.060724366	-2.820801061	-7.250288174
C	-19.967255857	-3.193831287	-6.241478562
H	-20.270756619	-3.594813943	-5.233352531
C	-20.661758541	-4.058559837	-7.306398248
H	-20.192952665	-5.057657137	-7.359293834
H	-20.539400039	-3.610866761	-8.309354214
C	-22.158475071	-4.179471675	-6.953878578
H	-22.537273444	-3.130266940	-6.799507455
C	-22.953645760	-4.803643806	-8.112492042
H	-22.940611550	-5.906977858	-8.046026624
H	-22.467075206	-4.557407158	-9.077114290
C	-24.406833451	-4.296456427	-8.133056992
H	-24.936990539	-4.654206456	-7.227336252
C	6.864208014	5.991042882	10.016656474
H	6.532012249	7.064976812	10.036803365
C	8.401867434	5.969559528	10.040276678
H	8.800394489	6.426128737	9.115227365
H	8.774264106	4.928376949	10.049960709
C	8.914229158	6.723992237	11.277677598
H	8.492038726	6.216904563	12.187708221
C	10.449091786	6.644946312	11.370585372
H	10.912984798	7.115091471	10.483933293
H	10.777536714	5.590135929	11.363672687
C	10.916551741	7.353774173	12.652077166
H	10.423530090	8.367072024	12.671034589
C	12.436717601	7.593190196	12.649061595
H	12.760246131	8.006599310	11.676876933
H	12.981325663	6.639886456	12.772877297
C	12.785598996	8.564572714	13.794952272
H	12.336124246	8.126025003	14.730255441

C	14.304087827	8.667516678	14.006232801
H	14.757652670	9.359396361	13.273977206
H	14.778088318	7.684132149	13.820975829
C	14.627568598	9.135097197	15.441993104
H	14.208638702	10.165539848	15.573168726
C	16.143910673	9.201688954	15.647727478
H	16.609538481	9.895117720	14.937247227
H	16.618692509	8.224273231	15.507077250
H	16.394971685	9.544273140	16.658063668
H	-24.947080755	-4.752918588	-8.988511670
C	-24.445622614	-2.800720873	-8.230240118
C	-23.992450162	-2.159155744	-9.385697293
C	-24.893331402	-2.037343361	-7.149159783
C	-23.972519666	-0.768695701	-9.451750421
H	-23.638109324	-2.748556764	-10.229684720
C	-24.878592134	-0.646435356	-7.220498903
H	-25.237977380	-2.529108794	-6.240133507
C	-24.415678573	-0.009701923	-8.370026385
H	-23.601294897	-0.272515340	-10.347431589
H	-25.219015342	-0.055160438	-6.370930059
H	-24.388057381	1.076855893	-8.418754828
C	-22.313417310	-4.951422993	-5.671262961
C	-21.853315229	-6.265766297	-5.558164324
C	-22.865359168	-4.319010302	-4.553970426
C	-21.924922255	-6.930651065	-4.337155363
H	-21.423141849	-6.767043652	-6.424041574
C	-22.937117254	-4.986114244	-3.333775134
H	-23.227485267	-3.294280216	-4.632218832
C	-22.463577466	-6.291493072	-3.221913524
H	-21.550148269	-7.949384595	-4.250853294
H	-23.354476729	-4.481781745	-2.463249033
H	-22.505721798	-6.807807229	-2.265772962
C	-20.402150774	-1.756694108	-6.357303479
C	-20.128453524	-1.013034350	-7.507387526
C	-21.085636698	-1.156974818	-5.296776100
C	-20.530989176	0.316668323	-7.593235044
H	-19.600490699	-1.474984132	-8.339514608
C	-21.487122362	0.173454778	-5.384353880
H	-21.305656455	-1.730935664	-4.397212146
C	-21.209572162	0.912794812	-6.531920129
H	-20.318864727	0.890073922	-8.493223593

H	-22.020218161	0.634742015	-4.555068145
H	-21.526662839	1.950037255	-6.603733007
C	-18.225734889	-3.399288040	-3.843254986
C	-17.840798743	-4.726353082	-3.640267264
C	-19.005558286	-2.754294201	-2.879862517
C	-18.227262609	-5.398088884	-2.483978908
H	-17.237021416	-5.236530478	-4.387627275
C	-19.397256148	-3.429866569	-1.727587366
H	-19.309736424	-1.718826100	-3.033160117
C	-19.011092111	-4.753577072	-1.528667626
H	-17.920156715	-6.429685125	-2.326463019
H	-20.007725946	-2.922188793	-0.983261397
H	-19.322715250	-5.283832893	-0.633006888
C	-16.203169213	-0.447139376	-3.889434717
C	-15.875183031	0.480812139	-4.880035174
C	-16.986877993	-0.047891015	-2.803783630
C	-16.323760461	1.794986379	-4.781711586
H	-15.265102864	0.176105955	-5.728127009
C	-17.435493800	1.266067125	-2.707867345
H	-17.243480969	-0.768718690	-2.026938923
C	-17.103320721	2.190142088	-3.696243695
H	-16.063340014	2.515350126	-5.554402269
H	-18.044217451	1.570224551	-1.858136308
H	-17.450066979	3.217212272	-3.620695253
C	-14.222975237	-1.983651528	-1.440502747
C	-14.036292239	-3.355611600	-1.259332692
C	-14.951630858	-1.257216480	-0.495004951
C	-14.587168697	-3.995359029	-0.152274959
H	-13.465399206	-3.926174952	-1.989382804
C	-15.497594914	-1.897701780	0.613571217
H	-15.094832602	-0.184785097	-0.629058034
C	-15.320021126	-3.269009483	0.784504745
H	-14.449348944	-5.066660139	-0.022087667
H	-16.065434969	-1.325568069	1.345165060
H	-15.753566047	-3.771912953	1.644671125
C	-12.192156907	0.926985153	-1.371057157
C	-12.010540645	1.777509153	-2.463871153
C	-12.898665521	1.382739280	-0.254804562
C	-12.543744515	3.063500607	-2.444928197
H	-11.456602944	1.431489205	-3.334413555
C	-13.425715881	2.670917212	-0.235229050

H	-13.036534008	0.726153030	0.604410825
C	-13.252429322	3.512334980	-1.332116380
H	-12.409551567	3.717901714	-3.303763549
H	-13.974648499	3.019125216	0.638031999
H	-13.669479121	4.515840405	-1.320942676
C	-10.146175424	-0.487850656	1.184219695
C	-9.936294338	-1.855139968	1.376947155
C	-10.848833204	0.242392581	2.146507947
C	-10.433606967	-2.485321599	2.514414539
H	-9.384839896	-2.429293073	0.634871681
C	-11.341548595	-0.388589022	3.285292885
H	-11.008421720	1.311668012	2.005941814
C	-11.137169326	-1.754344990	3.469785945
H	-10.273110763	-3.552021356	2.656778744
H	-11.886428035	0.187189986	4.031448753
H	-11.524930886	-2.248976389	4.356510694
C	-8.100586522	2.440813195	1.219532142
C	-7.882923197	3.297714829	0.138392864
C	-8.804063275	2.904974781	2.334219716
C	-8.371722712	4.600942179	0.169834263
H	-7.331504755	2.945095933	-0.731222075
C	-9.288867354	4.209450322	2.365361610
H	-8.970075719	2.243344459	3.184595251
C	-9.075757081	5.059006584	1.281842594
H	-8.203555598	5.262853037	-0.677281878
H	-9.833940180	4.564721758	3.238168528
H	-9.456748588	6.076635173	1.303306400
C	-6.050975763	0.979481117	3.752217504
C	-5.834268407	-0.386323794	3.947941364
C	-6.754825419	1.709227213	4.713797118
C	-6.324282796	-1.015420320	5.089089820
H	-5.282637414	-0.959906155	3.205500076
C	-7.241180612	1.079047076	5.855756586
H	-6.920776217	2.776990479	4.569875524
C	-7.029183579	-0.285056907	6.043973729
H	-6.156629038	-2.080576186	5.234736966
H	-7.787060664	1.654191362	6.601610799
H	-7.411984865	-0.778947202	6.933216067
C	-3.991122528	3.896865443	3.748493046
C	-3.777390825	4.746618640	2.660847680
C	-4.695713076	4.366184577	4.860198287

C	-4.272501110	6.047619963	2.682543802
H	-3.225896870	4.389318598	1.793269427
C	-5.186641430	5.668621816	4.881552988
H	-4.859850283	3.709481062	5.714780377
C	-4.978409914	6.510731002	3.791308434
H	-4.108696991	6.703646253	1.830005083
H	-5.733294851	6.027930737	5.751669620
H	-5.365401423	7.526076064	3.804748117
C	-1.936738714	2.426853421	6.270121726
C	-1.730243258	1.059694710	6.467829558
C	-2.643466699	3.161240181	7.226095120
C	-2.234546964	0.433820683	7.604504453
H	-1.176446505	0.482554745	5.729904085
C	-3.144484113	2.534135965	8.363401172
H	-2.801845779	4.230048367	7.080450507
C	-2.943433773	1.168608006	8.553065542
H	-2.075765187	-0.632572442	7.751179943
H	-3.693950472	3.112742263	9.103903201
H	-3.338275227	0.676834960	9.438085360
C	0.148915313	5.317691229	6.264664615
C	0.374741000	6.175983409	5.186194402
C	-0.560803724	5.780724048	7.375652575
C	-0.109812090	7.480699323	5.217997622
H	0.928375918	5.822676265	4.318330885
C	-1.041547343	7.086693625	7.407129248
H	-0.736737560	5.116355737	8.222038185
C	-0.818133420	7.938389160	6.327438455
H	0.064231932	8.143718011	4.373195318
H	-1.592340412	7.440578703	8.276802530
H	-1.195226491	8.957485530	6.349620043
C	2.243686857	3.883613609	8.763230104
C	2.506420189	2.531492162	8.993985475
C	1.517705165	4.613954177	9.707673161
C	2.045401355	1.917912065	10.155471891
H	3.072615060	1.956752773	8.263634714
C	1.059954343	3.999604683	10.869787410
H	1.311535897	5.670509449	9.534125399
C	1.322524941	2.650069756	11.095319335
H	2.250165066	0.863416025	10.329169546
H	0.496742944	4.575878861	11.601816639
H	0.965862592	2.169284199	12.002387069

C	4.272492904	6.791331006	8.717389752
C	4.494241174	7.658028293	7.644992324
C	3.532320046	7.233576699	9.817040134
C	3.971699363	8.948176094	7.669528401
H	5.072720530	7.321991064	6.786626671
C	3.012430817	8.524604489	9.840966465
H	3.359017513	6.562641872	10.658992661
C	3.229127192	9.383444405	8.765587830
H	4.140999287	9.617103999	6.828199256
H	2.434579929	8.860559851	10.700312796
H	2.819946247	10.390154957	8.780907103
C	6.300514923	5.271449794	11.213308139
C	6.511711240	3.903156474	11.397563962
C	5.534773964	5.980531269	12.142418009
C	5.947668241	3.249922268	12.489821884
H	7.113184379	3.346104876	10.681654066
C	4.972341984	5.325769833	13.234461108
H	5.372532976	7.050229912	12.008005974
C	5.174355894	3.958286664	13.407671349
H	6.108079482	2.182108316	12.624649964
H	4.371526050	5.883580894	13.950533352
H	4.728963038	3.444213790	14.255271919
C	8.460695529	8.160702843	11.248416572
C	8.858717734	9.020306112	10.222118313
C	7.639616813	8.642926193	12.270829439
C	8.441454678	10.348285225	10.221475258
H	9.500469329	8.651869261	9.423818744
C	7.221366120	9.970665795	12.267001950
H	7.327031154	7.977643674	13.075394151
C	7.621931314	10.825673924	11.242606111
H	8.761258870	11.016293366	9.424701256
H	6.585060336	10.340941768	13.068662688
H	7.302380295	11.864304112	11.242750182
C	10.498865022	6.577571294	13.872481441
C	9.600482426	7.151430756	14.775613221
C	10.996128751	5.295849308	14.117565628
C	9.205092385	6.451392163	15.912136571
H	9.207037075	8.150622840	14.590196827
C	10.600790644	4.598002818	15.255378857
H	11.697315124	4.842941871	13.419082706
C	9.704253286	5.173433577	16.153937124

H	8.507790854	6.905891269	16.613705118
H	10.997337768	3.602947021	15.446049856
H	9.400747108	4.628321107	17.043860133
C	12.158296337	9.906291727	13.528731375
C	12.492107935	10.647988507	12.392946118
C	11.177127613	10.387084923	14.399769941
C	11.837097668	11.846186618	12.122444261
H	13.255097724	10.280170804	11.708350875
C	10.523575978	11.585628865	14.126693039
H	10.910856430	9.814571311	15.287387922
C	10.848523704	12.314934761	12.985280685
H	12.090372365	12.412792504	11.228163579
H	9.747737647	11.946316811	14.801764602
H	10.326084460	13.241905839	12.762827068
C	13.988681199	8.200033914	16.437688067
C	13.000214851	8.680177303	17.300696539
C	14.324072202	6.843890043	16.463799778
C	12.351062539	7.811362995	18.174269386
H	12.721989631	9.732528643	17.278961634
C	13.668413815	5.976385673	17.332431086
H	15.087052613	6.460406748	15.788026002
C	12.679379930	6.457894054	18.187943802
H	11.576736566	8.190587727	18.840536126
H	13.920857536	4.916530571	17.336045392
H	12.155327494	5.776363308	18.854219253

Final heat of formation

212.78699 kcal/mol

Geometry optimization
Converged to a stationary point.
